# Beneficial Effects of Probiotic *Bifidobacterium longum* in a Lithium–Pilocarpine Model of Temporal Lobe Epilepsy in Rats

**DOI:** 10.3390/ijms24098451

**Published:** 2023-05-08

**Authors:** Olga E. Zubareva, Alexandra V. Dyomina, Anna A. Kovalenko, Anna I. Roginskaya, Tigran B. Melik-Kasumov, Marina A. Korneeva, Alesya V. Chuprina, Alesya A. Zhabinskaya, Stepan A. Kolyhan, Maria V. Zakharova, Marusya O. Gryaznova, Aleksey V. Zaitsev

**Affiliations:** 1 Sechenov Institute of Evolutionary Physiology and Biochemistry, Russian Academy of Sciences, 194223 Saint Petersburg, Russia; zubarevaoe@mail.ru (O.E.Z.);; 2 Institute of Physiology of the National Academy of Sciences of Belarus, 220072 Minsk, Belarus; tigranbmk@gmail.com (T.B.M.-K.);

**Keywords:** neuroinflammation, interleukin-1β, interleukin-1 receptor antagonist, social behavior, memory test, anxiety, astrogliosis, microgliosis, neuronal loss, peroxisome proliferator-activated receptor

## Abstract

Epilepsy is a challenging brain disorder that is often difficult to treat with conventional therapies. The gut microbiota has been shown to play an important role in the development of neuropsychiatric disorders, including epilepsy. In this study, the effects of *Bifidobacterium longum*, a probiotic, on inflammation, neuronal degeneration, and behavior are evaluated in a lithium–pilocarpine model of temporal lobe epilepsy (TLE) induced in young adult rats. *B. longum* was administered orally at a dose of 10^9^ CFU/rat for 30 days after pilocarpine injection. The results show that *B. longum* treatment has beneficial effects on the TLE-induced changes in anxiety levels, neuronal death in the amygdala, and body weight recovery. In addition, *B. longum* increased the expression of anti-inflammatory and neuroprotective genes, such as *Il1rn* and *Pparg*. However, the probiotic had little effect on TLE-induced astrogliosis and microgliosis and did not reduce neuronal death in the hippocampus and temporal cortex. The study suggests that *B. longum* may have a beneficial effect on TLE and may provide valuable insights into the role of gut bacteria in epileptogenesis. In addition, the results show that *B. longum* may be a promising drug for the comprehensive treatment of epilepsy.

## 1. Introduction

Temporal lobe epilepsy (TLE) is one of the most common and difficult-to-treat neurological disorders. The global prevalence of epilepsy is approximately 6.38 per 1000 people [1]. This pathology develops spontaneous recurrent seizures (SRSs) associated with neurodegeneration, astrogliosis and microgliosis, and the reorganization of neuronal circuits [2,3,4]. TLE can lead to psychological problems, including impaired memory [5], social interaction [6], anxiety, and depression [7]. Approximately one third of epilepsies remain drug-resistant, despite the availability of a large number of anticonvulsants [8]. At the same time, many antiepileptic drugs have negative side effects, such as dizziness, drowsiness, and slowed thinking [9]. In addition, existing drugs can prevent seizures, but do not prevent epileptogenesis [10]. Taken together, this makes the search for new effective therapies urgent.

In recent years, the role of the gut–brain axis, diet, and gut microbiota in the pathogenesis of neurological and psychiatric disorders [11,12,13,14], including epilepsy [15,16,17,18], has been intensively studied. It has been shown that seizure frequency is increased in patients with a comorbidity of epilepsy and irritable bowel syndrome [19], which is associated with changes in the balance of the gut microbiota [20]. The composition of the gut microbiota changes significantly in patients with pharmacoresistant epilepsy, but not in those with drug-sensitive epilepsy [21]. The former have a significant increase in the species diversity of the microbiota (α-diversity), an increase in the content of rare representatives [22]. In drug-sensitive epilepsy, levels of bifidobacteria and lactobacilli are correlated with seizure frequency, being higher in patients with four or fewer seizures per year than in those with more frequent seizures [23]. Significant changes in the composition of the fecal microbiota in patients with epilepsy have also been shown [22,24,25,26,27]. Alternatively, a significant improvement in the quality of life and reduction in seizure frequency was observed in 28.9% of patients with drug-resistant epilepsy who received a mixture of eight bacterial species [28]. Probiotics can alleviate behavioral disturbances, including anxiety, depressive-like behavior, and cognitive impairment [29], which are common in patients with epilepsy [5,30] and animals in experimental models [31,32,33]. Probiotics reduced seizure severity and partially improved spatial learning and memory in the pentylenetetrazole-induced kindling model in rats [31].

Microbiota may have neuroprotective effects in epilepsy due to its modulatory effects on neuroimmune interactions and neuroinflammation [34], which plays an important role in the pathogenesis of epilepsy [35,36]. Neuroinflammation is mainly associated with the increased activation of microglia and astroglia cells, which produce proinflammatory cytokines, such as interleukin-1β (IL-1β) and others [37]. Proinflammatory cytokine production is increased in the brain in epilepsy cases [38]. The effect of proinflammatory cytokines is limited by anti-inflammatory cytokines, particularly interleukin-1 receptor antagonists (IL-1ra) [39]. The IL-1ra/IL-1β ratio may be one of the physiological mechanisms controlling seizures [40]. It has been suggested that certain probiotics may increase the production of anti-inflammatory cytokines in the brain, thereby mitigating the neural and behavioral effects of neuroinflammation [41].

Another possible mechanism of the neuroprotective action of probiotics is an increase in the expression of peroxisome proliferator-activated receptors (PPARs) in brain cells. PPARs (PPARα, PPARβ/δ, PPARγ) are nuclear transcription factors that regulate the expression of a number of genes involved in carbohydrate and lipid metabolism, the development of inflammatory responses, cell differentiation, and apoptosis [42,43]. These receptors are one of the key links in gut–brain interactions [44,45]. The ligands of these receptors are short-chain fatty acids secreted by the gut microbiota, which play a neuroprotective role in epilepsy cases [46].

As described above, in most of the works, both in animal models and patients with epilepsy, the mixture of several different probiotic bacteria (*Bifidobacteria*, *Lactobacilli*, *Enterococci*, *Streptococci*, etc.) with the addition of prebiotics is usually used to assess their effects on the course of epilepsy. Among others, probiotic mixtures containing *Lactobacillus* and *Bifidobacterium* spp. have been found to have the most pronounced positive effects on the course of epilepsy [47]. However, the question of whether monoprobiotic treatment can influence epileptogenesis remains open. The mechanisms of the neuroprotective effects of probiotics in epilepsy are also poorly understood.

The aim of this work is to investigate the neuroprotective, anti-inflammatory, and behavioral effects of a 30-day treatment with the probiotic *B. longum* in a lithium-pilocarpine model of TLE. The choice of *B. longum* as the object of study, among other probiotics used in mixtures, was based on the fact that it is a psychobiotic, i.e., a probiotic with a pronounced effect on the central nervous system. Its effects on stress-related behavior, physiology, and cognitive abilities have been previously demonstrated [48].

## 2. Results

### 2.1. Dynamics of Body Weight, Survival, and SRSs

Body weight is an integrative indicator of the health status of experimental animals. The lithium–pilocarpine model is characterized by a significant decrease in body weight after pilocarpine administration, followed by a gradual recovery of body weight, which is quicker in rats with a less severe pathological process. Therefore, we investigated how body weight changes after pilocarpine administration; measurements were taken during the month in which the animals were given the probiotic.

**Figure 1:**
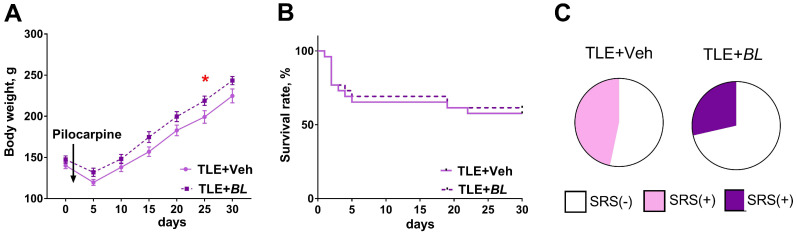
Effects of *B. longum* treatment on body weight dynamics, survival rate, and spontaneous recurrent seizures (SRSs) of TLE rats. (**A**) Body weight dynamics of TLE rats with *B. longum* treatment (*BL*) and without treatment (Veh). * *p* < 0.05 post hoc Sidak comparison test. Data are expressed as mean and standard error of the mean. (**B**) Survival of animals during the 30-day course of administration of probiotic. (**C**) Percentage of rats with SRSs in groups of treated (TLE + *BL*) and untreated (TLE + Veh) animals.

**Figure 2:**
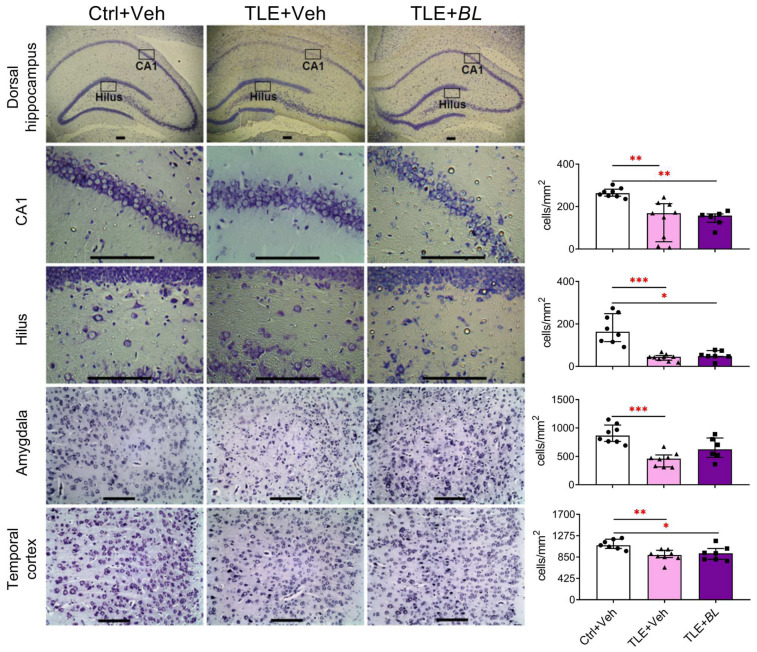
Effects of 30 days of *B. longum* (*BL*) administration on neuronal density in the dorsal hippocampus, temporal cortex, and amygdala. Scale bar = 150 µm. Ctrl—control rats; TLE + Veh—untreated TLE rats; TLE + *BL*—*B. longum*-treated TLE rats. Kruskal–Wallis H test followed by Dunn’s multiple comparison test: * *p* < 0.05; ** *p* < 0.01, *** *p* < 0.001. Data are presented as median and interquartile range. Each data point corresponds to a single animal.

**Figure A1:**
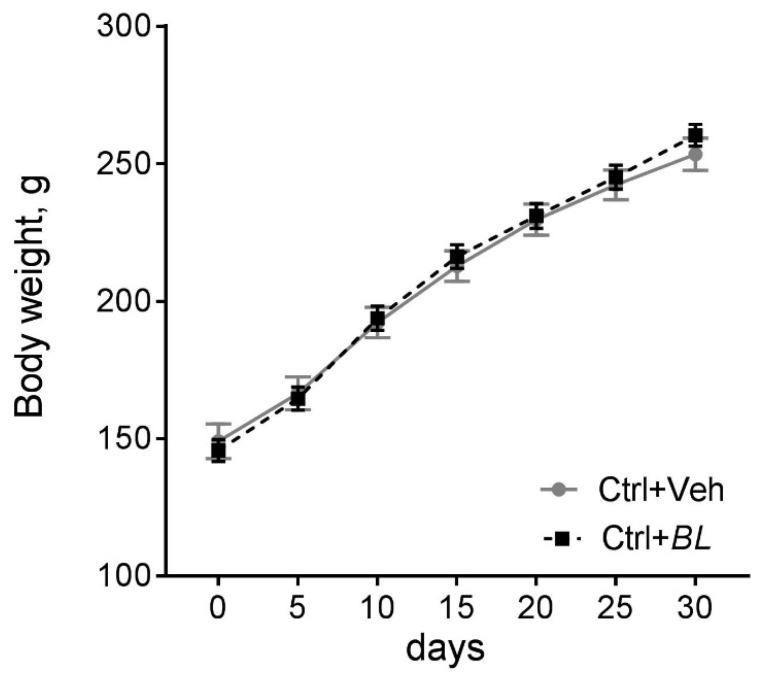
Effects of *B. longum* treatment on body weight dynamics of control rats after administration of *B. longum* (*BL*, *n* = 18) or water (Veh, *n* = 19). A two-way mixed ANOVA model: F[Time × *BL*]_6210_ = 1.17; *p* = 0.32; F[Time]_6210_ = 663; *p* < 0.001; F[*BL*]_1,35_ = 0.56; *p* = 0.81. Data are presented as means and standard errors of the means.

Rats in all four groups did not differ in body weight before pilocarpine injection (a one-way ANOVA: F_3,65_ = 0.60; *p* = 0.62). Probiotic administration had no effect on the weight dynamics of the control animals (Appendix A, Figure A1). During the first few days after pilocarpine-induced seizures, TLE animals lost weight, with a maximum reduction of 24.0 ± 1.1% of their initial body weight (Figure 1A). Their body weight began to recover one week after the seizures (F[Time]_6,180_ = 252; *p* < 0.0001). The weight of probiotic-treated rats was higher than that of the control animals (Figure 2A, F[*BL*]_1,30_ = 4.6; *p* = 0.04). The differences became more evident after 20 days of treatment, when the cumulative effect of *B. longum* is likely to be manifested.

At the same time, the use of the probiotic had no effect on the survival rate of the rats (Figure 1B, log-rank Mantel–Cox test—χ^2^ = 0.08; *p* = 0.78). The mortality rate was 40% in the TLE + Veh group and 37% in the TLE + *BL* group (Fisher’s exact test *p* = 0.52). It should be noted that the main mortality rate was observed in the first days after pilocarpine administration, i.e., before the effects of the probiotic began to show in the body weight dynamics.

At 2 months after pilocarpine administration, SRSs were observed in 47% of untreated and 29% of treated rats within the video recording period (Figure 1C, Fisher’s exact test *p* = 0.45).

### 2.2. Probiotic Treatment Reduces Neuronal Loss in the Amygdala, but Not in the Hippocampus or Temporal Neocortex, in Rats with TLE

The lithium–pilocarpine model of TLE is characterized by significant neuronal cell loss in the temporal lobe structures [49,50]. We tested whether probiotic treatment would have a neuroprotective effect. We counted and compared the density of cells in the hippocampus, temporal cortex, and amygdala 30 days after pilocarpine-induced SE in three rat groups (Figure 2). We found neuronal losses in TLE rats in all the brain structures examined. The most dramatic changes (2–4-fold) in the neuronal density between the control and TLE rats were found in the hilus (H = 16.4; *p* < 0.001) and CA1 regions (H = 15.0; *p* < 0.001) of the hippocampus and amygdala (H = 13.7; *p* < 0.001). Some samples contained only a few cells in hippocampal areas. A less pronounced decrease in neuronal density (approximately 25%) was observed in the temporal cortex (H = 10.0; *p* < 0.01).

The neuroprotective effect of the probiotic was rather weak. We found that probiotic administration attenuated neuronal death in the amygdala (Dunn’s test vs. control, *p* > 0.05), but did not significantly affect neurodegeneration in the hippocampus and temporal cortex (Figure 2).

### 2.3. The Expression of Anti-Inflammatory and Neuroprotective Genes in Brain Cells Is Increased after 30 Days of Treatment with B. longum

**Figure 3:**
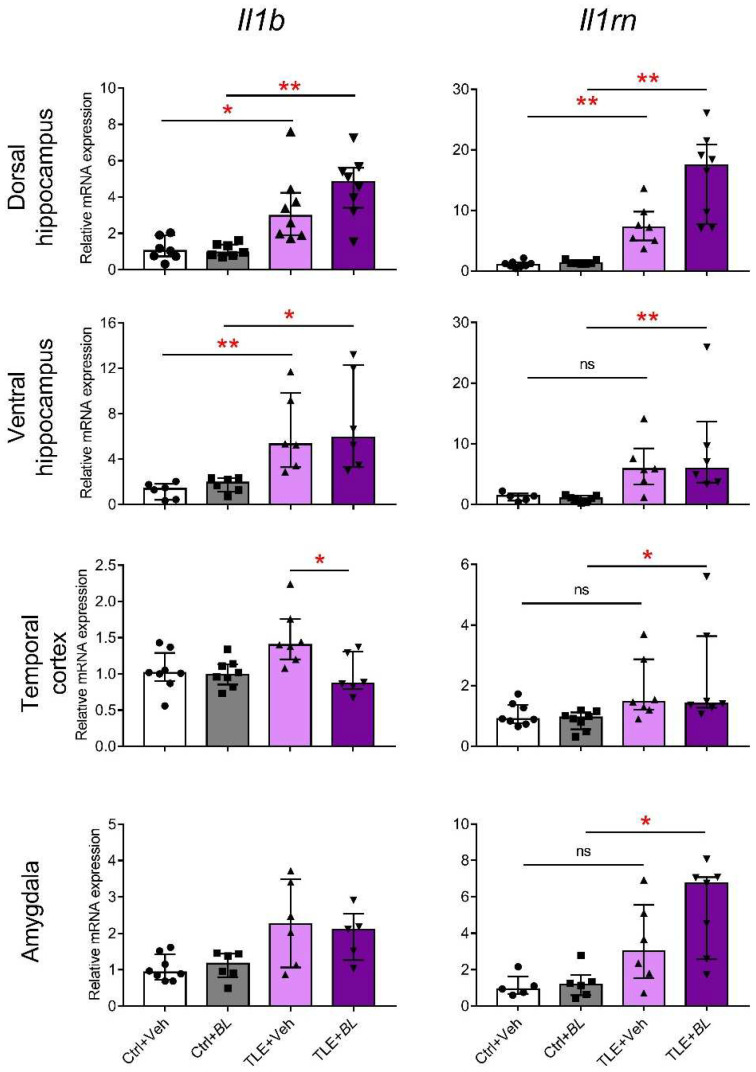
Effects of 30-day *B. longum* (*BL*) treatment on relative *Il1b* and *Il1rn* mRNA expressions in the rat brain structures of control (Ctrl) or TLE rats. Kruskal–Wallis H test followed by Dunn’s multiple comparison test: * *p* < 0.05; ** *p* < 0.01; ns—not statistically significant. Data are presented as median and interquartile range. Each data point corresponds to a single animal.

Given the important role of neuroinflammation in epileptogenesis [38] and the previously demonstrated ability of *B. longum* to suppress neuroinflammatory processes [51], we examined the expression of proinflammatory and anti-inflammatory cytokine genes (*Il1b* and *Il1rn*, respectively) in the brains of TLE and control animals (Figure 3).

A group comparison using the Kruskal–Wallis H-criterion showed that *Il1b* gene expression was significantly altered in TLE rats in all the examined structures: dorsal hippocampus (H = 19.5; *p* < 0.01), ventral hippocampus (H = 17.8; *p* < 0.01), temporal cortex (H = 10.7; *p* < 0.05), and amygdala (H = 8.5; *p* < 0.05). However, intergroup comparisons between probiotic untreated control and TLE rats with Dunn’s multiple comparison test showed significant differences only in the ventral (*p* < 0.01) and dorsal (*p* < 0.05) parts of hippocampus. Probiotic treatment did not affect the level of *Il1b* mRNA in the ventral and dorsal parts of hippocampus and amygdala, but attenuated the expression of this gene in the temporal cortex of TLE rats (*p* < 0.05).

The Kruskal–Wallis test showed that *Il1rn* mRNA expression was altered in the TLE groups in all brain regions examined: dorsal hippocampus (H = 23.4; *p* < 0.001), ventral hippocampus (H = 15.2; *p* < 0.01), temporal cortex (H = 12.7; *p* < 0.01), and amygdala (H = 12.5; *p* < 0.05). However, post hoc tests revealed a significant increase in the TLE + Veh group compared to the Ctrl + Veh group, only in the dorsal hippocampus, whereas in the probiotic-treated animals, a significant increase in expression (Ctrl + *BL* vs. TLE + *BL*) was observed in all brain structures examined. Thus, the results suggest the ability of probiotics to somewhat limit neuroinflammatory and enhance anti-inflammatory processes in the TLE.

We also tested the hypothesis that the protective and anti-inflammatory effects of the probiotic may be related to its effect on the expression in the brain of neuroprotective PPARs genes, which play an important role in the gut–brain interaction [44].

**Figure 4:**
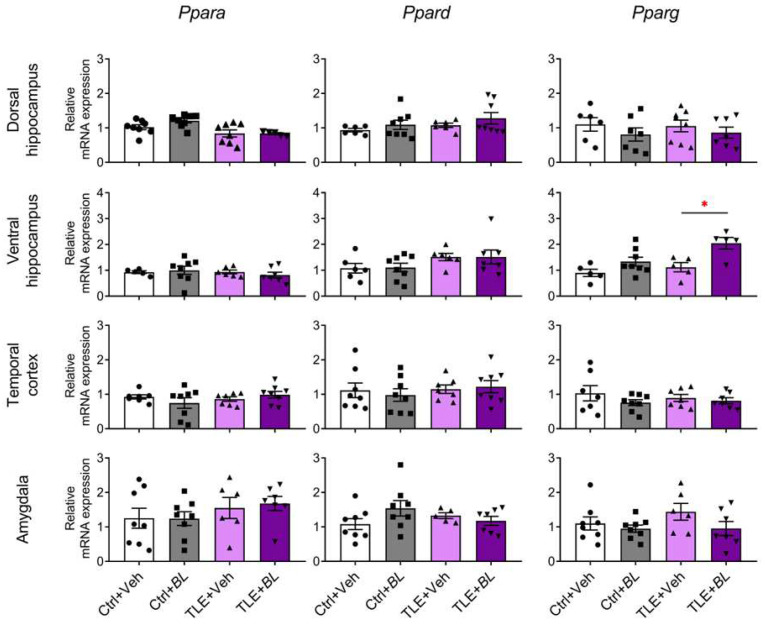
The effects of a 30-day course of *B. longum* (*BL*) or water (Veh) administration on the relative expression of *Ppars* genes in rat brain structures of control (Ctrl) and epileptic (TLE) rats. Two-way ANOVA followed by post hoc Sidak test: * *p* < 0.05. Data are presented as means and standard errors of the mean. Each data point represents the result of a single animal. The full result of the statistical processing can be found in Appendix B (Table A1).

*Ppars* gene expression was almost unchanged in epilepsy cases (Figure 4). A two-way ANOVA revealed only a decrease in PPARα (gene—*Ppara*) expression in the dorsal hippocampus of epileptic rats (F[TLE]_1,25_ = 12, *p* < 0.01) and a decreased expression of PPARβ/δ (gene—*Ppard*) and PPARγ (gene—*Pparg)* genes in the ventral hippocampus (F[TLE]_1,23_ = 4.3, *p* = 0.05 and F[TLE]_1,19_ = 6.2, *p* = 0.02, respectively).

Probiotic administration also had almost no effect on *Ppar* gene expression, except for the *Pparg* gene in the ventral hippocampus (Figure 4). Its expression was increased in *B. longum*-treated rats (F[*BL*]_1,19_ = 13.3, *p* < 0.01), and this effect was more pronounced in epileptic animals (TLE + *BL* group vs. TLE + Veh group, Sidak post hoc test, *p* < 0.05).

### 2.4. Probiotic Treatment Does Not Significantly Affect Astrogliosis and Microgliosis in Temporal Lobe Structures in Rats with TLE

Neuroinflammation is associated with the activation of glial cells, primarily microglia and astroglia. In the lithium–pilocarpine rat model of TLE, astrocytes in the hippocampus and temporal cortex are activated, as evidenced by hypertrophy of cell bodies and processes as detected by glial fibrillary acidic protein (GFAP) immunostaining [52,53]. Microgliosis persists for at least 30 days after seizure induction and correlates with local neuronal death in the pilocarpine rodent TLE model [54,55]. It is likely that alterations in glial function may play an important role in epileptogenesis [56,57,58]. Therefore, we tested how probiotic treatment would affect astrogliosis and microgliosis in the hippocampus, temporal cortex, and amygdala. To this end, we first examined the changes in the expression of *Aif1* (peptide—IBA1) and *Gfap* genes as markers of microglial and astrocytic cell activation in our experiment.

**Figure 5:**
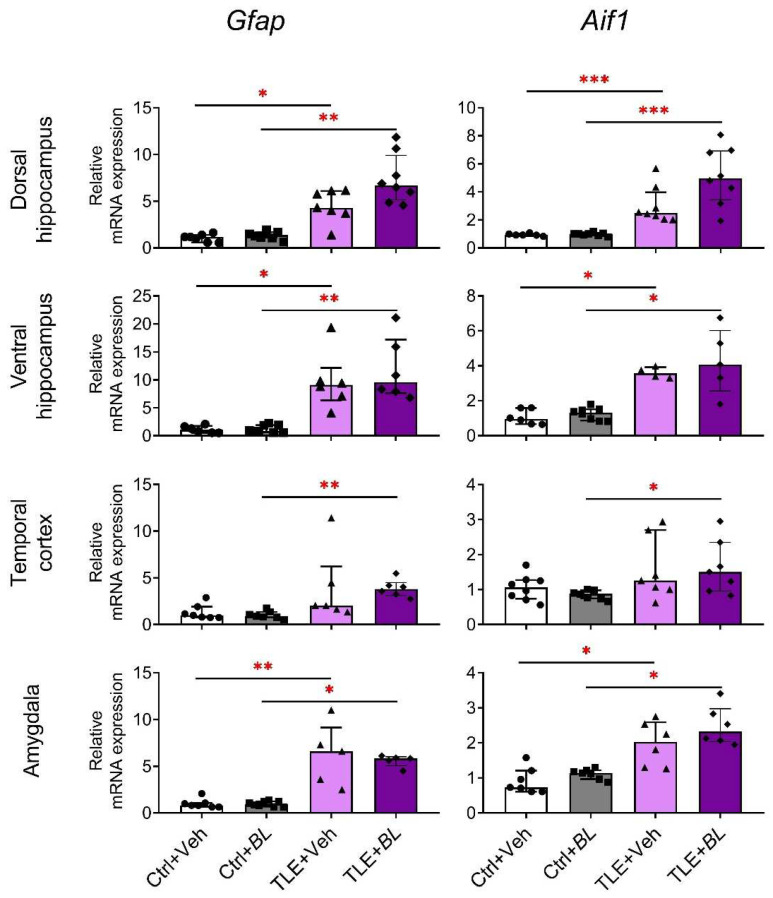
The relative *Gfap* and *Aif1* mRNA expressions in the rat brain structures of control (Ctrl) or epilepsy (TLE) groups that received water (Veh) or *B. longum* (*BL*). Kruskal–Wallis H test followed by Dunn’s multiple comparison test. Dorsal hippocampus (*Gfap*—H = 21.9; *p* < 0.001; *Aif1*—H = 22.9; *p* < 0.001), ventral hippocampus (*Gfap*—H = 18.8; *p* < 0.001; *Aif1*—H = 16.0; *p* < 0.001), temporal cortex (*Gfap*—H = 15.3; *p* < 0.001; *Aif1*—H = 8.0; *p* < 0.05), and amygdala (*Gfap*—H = 17.4; *p* < 0.001; *Aif1*—H = 18.2; *p* < 0.001). Dunn’s multiple comparison test: * *p* < 0.05, ** *p* < 0.01, *** *p* < 0.001. Data are presented as median and interquartile range. Each data point represents the result of a single animal.

Our results show that *Gfap* and *Aif1* mRNA expressions increase in all brain structures of TLE rats (Figure 5). However, the comparison of Ctrl + Veh and TLE + Veh groups showed no significant difference in the temporal cortex. Probiotic treatment not only failed to block this effect, but also caused *Gfap* and *Aif1* to be more upregulated. Therefore, the increased expression of these genes was observed in all brain regions examined in the TLE + *BL* group compared to the Ctrl + *BL* group, including the temporal cortex.

**Figure 6:**
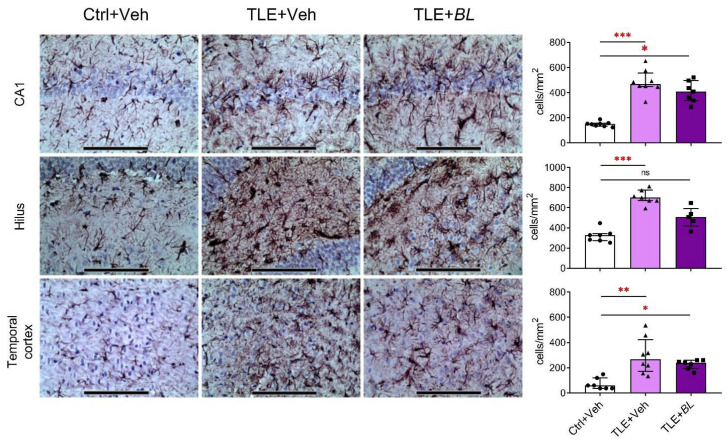
Effects of a 30-day course of *B. longum* (*BL*) administration on astroglial density (immunohistochemical staining for GFAP) in CA1, hilus, and temporal cortex. Micrographs were taken from animals with values near the median. Scale bar = 150 µm. Ctrl + Veh—control rats; TLE + Veh—untreated TLE rats; TLE + *BL*—probiotic-treated TLE rats. Kruskal–Wallis H test: CA1—H = 15.9; *p* < 0.001; hilus—H = 15.2; *p* < 0.001; temporal cortex—H = 13.4; *p* < 0.001. Dunn’s multiple comparison test: ns—not statistically significant, * *p* < 0.05, ** *p* < 0.01, *** *p* < 0.001. Data are presented as median and interquartile range. Each data point represents the result of a single animal.

These RT-PCR results were confirmed by immunohistochemistry. We observed increased numbers of GFAP-positive cells in the hippocampus and temporal cortex of epileptic rats (Figure 6). The number of astrocytes was significantly higher than in the control group in all regions (CA1 area: 3-fold increase, Dunn’s test, *p* < 0.001; hilus: 2-fold increase, *p* < 0.001; temporal cortex: 4-fold increase, *p* < 0.01). *B. longum* treatment did not reduce astroglia cell density in the CA1 and temporal cortex. These density values were significantly higher than in the control group (Dunn’s test, *p* < 0.05). However, in the hilus of probiotic-treated TLE rats, the density of GPAP-positive cells was not significantly different from the controls (*p* > 0.05).

**Figure 7:**
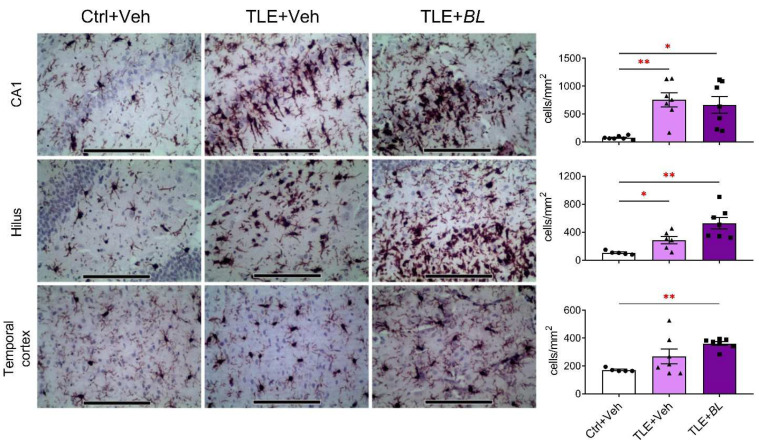
The effects of a 30-day course of *B. longum* (*BL*) or water (Veh) administration on the density of microglia (immunohistochemical staining for IBA1) in the CA1, hilus, and temporal cortex. Micrographs were taken from animals with values near the median. Scale bar = 150 µm. Ctrl + Veh—control rats; TLE + Veh—untreated TLE rats; TLE + *BL*—probiotic-treated TLE rats. Welch’s ANOVA: CA1: F_2,8.1_ = 20.2; *p* = 0.001; hilus: F_2,7.8_ = 17.1; *p* = 0.001; temporal cortex: F_2,9.1_ = 70.2; *p* < 0.001. Games–Howell post hoc test: * *p* < 0.05, ** *p* < 0.01. Data are presented as means and standard errors of the mean. Each data point represents the result of a single animal.

In the TLE rats, compared to the controls, the density of IBA1-immunopositive cells was increased in the CA1 area, hilus, and temporal cortex (Figure 7). However, when comparing the Ctrl + Veh and TLE + Veh groups, significant changes were only observed in the hippocampus (CA1: 10-fold increase; Games–Howell test, *p* < 0.01; hilus: 3-fold increase; *p* < 0.05), but not in the temporal cortex (*p* > 0.05). Probiotics did not reduce the number of IBA1-immunopositive cells in any of the structures examined; on the contrary, the increase in the number of IBA1-immunopositive cells was more pronounced in the hilus and temporal cortex of *B. longum*-treated TLE rats (*p* < 0.01).

Overall, it can be concluded that the probiotic did not block the development of astrogliosis and microgliosis in the rat TLE model.

### 2.5. Effects of B. longum Treatment on the Behavior of TLE and Control Rats

In this study, we sought to determine whether *B. longum* alleviates the behavioral deficits characteristic of TLE animals.

#### 2.5.1. Motor Hyperactivity

**Figure 8:**
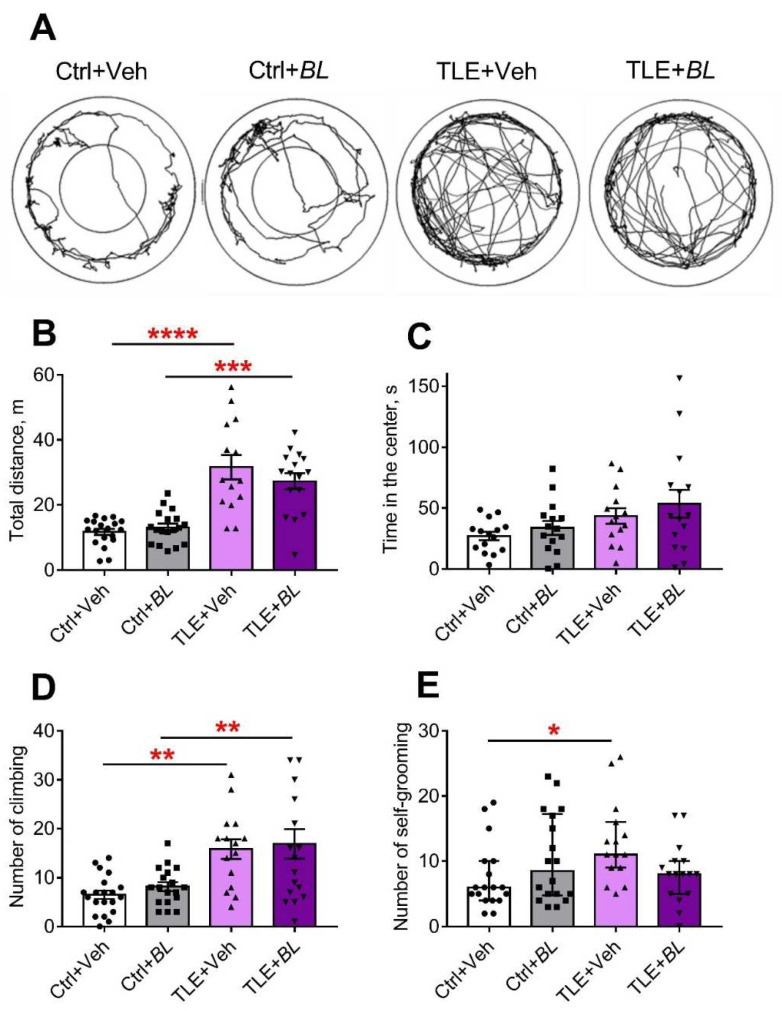
The effects of a 30-day course of *B. longum* (*BL*) or water (Veh) administration on the behavior of control (Ctrl) and TLE rats in the OFT. (**A**) Track samples; (**B**) total distance; (**C**) time in the center; (**D**) number of climbs. Two-way ANOVA followed by post hoc Sidak test: ** *p* < 0.01, *** *p* < 0.001, **** *p* < 0.0001. Data are presented as means and standard errors of the mean. (**E**) Number of self-grooming episodes. Kruskal–Wallis H test followed by Dunn’s multiple comparison tests. Data are presented as median and interquartile range. * *p* < 0.05. Each data point represents the result of a single animal.

Hyperactivity is one of the hallmarks of animal behavior in the lithium–pilocarpine model of TLE [59,60]. We assessed the horizontal and vertical activity of the rats in the open field test (OFT), analyzing the length of distance traveled and climbing while exploring a novel space (Figure 8A–D). Horizontal activity increased even more in animals with TLE (Figure 8B). The distance traveled was 2.7 times longer in the TLE + Veh group and 2.2 times higher in the TLE + *BL* group, compared to the corresponding control (F[TLE]_1,62_ = 66, *p* < 0.001). *B. longum* did not block this hyperactivity (Figure 8A,B; F[*BL*]_1,62_ = 0.26; *p* = 0.61; F[TLE × *BL*]_1,62_ = 1.36; *p* = 0.25).

The number of climbs was more than twice as high in the TLE groups compared to the controls (F[TLE]_1,65_ = 25.5, *p* < 0.001). Probiotic administration did not prevent these changes (Figure 8D; F[*BL*]_1,65_ = 0.58, *p* = 0.36; F[TLE × *BL*]_1,65_ = 0.001, *p* = 0.98). Climbing time showed similar differences between the groups (Appendix B, Table A2).

Thus, TLE rats exhibited increased motor activity in the OFT and probiotic treatment did not reduce it.

#### 2.5.2. Anxiety

**Figure 9:**
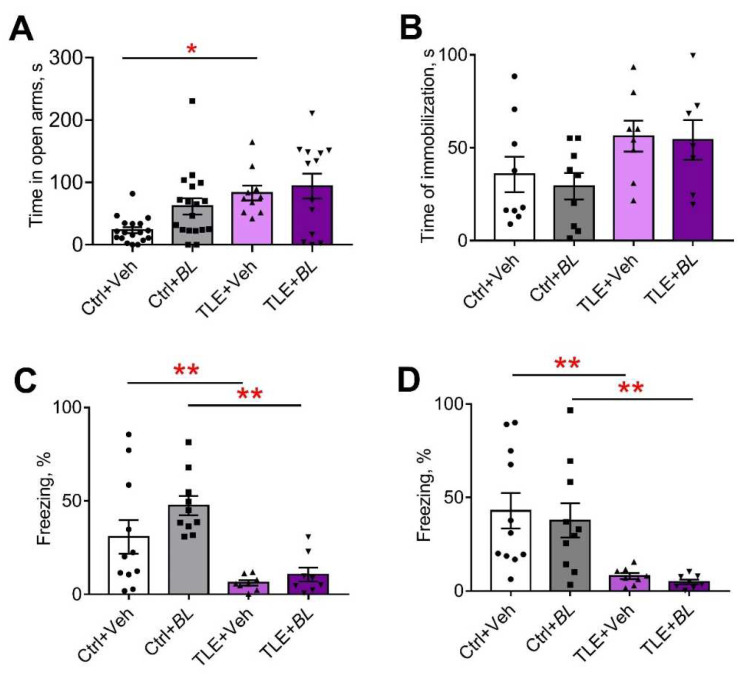
The effects of a 30-day course of *B. longum* (*BL*) or water (Veh) administration on the behavior of control (Ctrl) and TLE rats in the elevated plus maze (EPM), forced swim test (FST), and fear conditioning test (FCT): (**A**) time in the open arms of the EPM; (**B**) time of immobilization in the FST; (**C**) time of freezing in the familiar cage in the FCT; (**D**) time of freezing in a novel cage during tone presentation. Two-way ANOVA followed by post hoc Sidak test: * *p* < 0.05, ** *p* < 0.01. Data are presented as means and standard errors of the mean. Each data point represents the result of a single animal.

**Figure 10:**
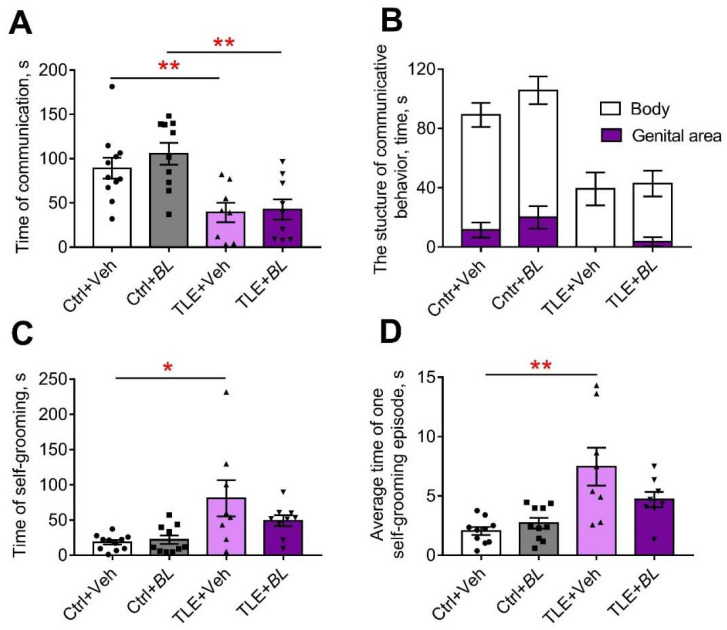
The effects of a 30-day course of *B. longum* (*BL*) or water (Veh) administration on the behavior of control (Ctrl) and TLE rats in the social interaction test (SIT): (**A**) time of communication; (**B**) structure of communicative behavior; (**C**) total time of self-grooming; (**D**) average time of a self-grooming episode. Two-way ANOVA followed by post hoc Sidak test: * *p* < 0.05, ** *p* < 0.01. Data are presented as means and standard errors of the mean. Each data point represents the result of a single animal.

The level of anxiety was assessed by several parameters obtained in the OFT (Figure 8C,E and Appendix B, Table A2 and Table A3), the elevated plus maze (EPM) (Figure 9A and Appendix B, Table A3), and the social interaction test (SIT) (Figure 10C,D, Table A5). All indicators suggest that the level of anxiety was altered in the TLE rats.

The TLE rats did not avoid open areas; they spent more time in the central part of the OFT (Figure 8C, F[TLE]_1,55_ = 6.1; *p* = 0.02). However, no significant differences were observed by the pairwise comparison. Probiotic treatment had no significant effect on this parameter (F[*BL*]_1,55_ = 1.3; *p* = 0.26; F[TLE × *BL*]_1,55_ = 0.05; *p* = 0.82).

In the EPM, almost one third of the TLE animals (5 out of 16) jumped out of the open arms of the maze, which was never observed in the control animals. Some of the probiotic-treated TLE rats (3 out of 17) also jumped out of the open arms. The remaining TLE rats spent more time in the open arms of the maze than the controls (Figure 9A; F[TLE]_1,54_ = 10.8; *p* = 0.002). Probiotic treatment had no effect on this parameter (F[*BL*]_1,54_ = 2.84; *p* = 0.10; F[TLE × *BL*]_1,54_ = 1.48; *p* = 0.23).

The change in the level of anxiety was also assessed by self-grooming episodes [61]. We found an increase in the number of self-grooming episodes in TLE rats compared to the controls in the OFT (Figure 8E,H = 8.3; *p* < 0.05). However, significant differences were observed only between the untreated groups (TLE + Veh vs. Ctrl + Veh); the use of probiotics abolished the difference between the groups.

Similar changes were seen in the SIT (Figure 10C,D, Table A5). Untreated TLE rats showed an increase in total grooming time (F[TLE]_1,34_ = 13.8; *p* < 0.001) and an increase in the average duration of grooming episodes (F[TLE]_1,32_ = 20.24; *p* < 0.001). No such changes were observed in the treated animals.

Thus, the anxiety level of rats in the TLE model changed and probiotic treatment levels these changes, with the effect lasting for at least one month after treatment.

#### 2.5.3. Depressive-like Behavior

Immobilization time in the forced swim test (FST) was used as an indicator of depressive-like behavior (Figure 9B). TLE increased depressive-like symptoms (F[TLE]_1,29_ = 6.5, *p* < 0.05). However, there were no significant differences between the groups. We observed no effect of probiotics on the depressive-like behavior ([*BL*]_1,29_ = 0.22; *p* = 0.64; F[TLE × *BL*]_1,29_ = 0.06; *p* = 0.81).

#### 2.5.4. Memory

We previously observed memory deficits in rats in a lithium–pilocarpine model of TLE [59,60]. This study obtained similar results with the fear conditioning test (FCT). TLE rats showed impairments in their contextual memory (decreased freezing time in the familiar cage compared to the controls; Figure 9C; F[TLE]_1,32_ = 23.1, *p* < 0.001) and memory for conditioned stimuli (decreased response to tone in a novel cage, Figure 9D; F[TLE]_1,32_ = 19.5, *p* < 0.001). Both untreated and treated TLE rats showed these memory deficits (Table A4). Thus, probiotic treatment had no effect on memory impairment.

#### 2.5.5. Social Behavior

Another behavioral feature observed in TLE rats was the severe impairment of communicative activity in the SIT (F[TLE]_1,34_ = 6.8, *p* < 0.01, Figure 10A; Table A5). *B. longum* treatment was unable to prevent this impairment and a more than 2-fold reduction in communication time was observed in both treated and untreated TLE animals compared to the controls.

The structure of communicative behavior was altered in TLE rats (Figure 10B). Control rats actively sniffed not only the body, but also the genital area of the unfamiliar male. This behavior completely disappeared in untreated TLE rats. It was present in some probiotic-treated TLE animals, perhaps an indication of their more confident behavior.

## 3. Discussion

In this study, we investigated the effects of a 30-day treatment with probiotic *B. longum* in a rat model of TLE. In this TLE model, neuronal death, astrogliosis, microgliosis, increased expression of proinflammatory mediators in brain structures, and behavioral disturbances (hyperactivity, social and memory deficits, and changes in anxiety levels) were observed. Probiotic treatment had some beneficial effects in the TLE model. Probiotic treatment (1) promoted the recovery of body weight reduced after pilocarpine-induced epileptic status, (2) suppressed the TLE-induced increase in pro-inflammatory cytokine *Il1b* gene expression in the temporal cortex and increased the expression of anti-inflammatory cytokine *Il1rn* in several brain regions studied, (3) reduced neuronal loss in the amygdala, and (4) attenuated TLE-induced anxiety changes in rats. However, the probiotic did not alleviate most of the other negative effects of TLE, such as hyperactivity, depressive-like behavior, impaired social behavior, and memory deficits.

We chose *B. longum* for this study because of its neuroprotective effects on the nervous system, which have previously been shown in humans with stress and anxiety symptoms [62] and in experimental models of stress and depression [63]. *B. longum* is a Gram-positive, catalase-negative, bacillus that is normally present in the human gastrointestinal tract, colonizing it as early as infancy [64]. *B. longum* is also part of the normal gut microbiota of rats [65]; therefore, data from the experimental model we used can be extrapolated to humans.

The reduction in body weight that occurred after pilocarpine-induced seizures may be related, at least in part, to the development of neuroinflammation. It is known that one of the effects of increased levels of proinflammatory cytokines in the blood and brain is the suppression of hunger and reduction in body weight [66,67]. We did not examine the eating behavior of the experimental animals in our work; however, given the anti-inflammatory effects of the probiotic, we can assume that its administration may have a positive effect on food motivation in TLE animals.

In this study, using a lithium–pilocarpine model of TLE, we showed, for the first time, that a 30-day course of *B. longum* administration reduced the expression of the proinflammatory cytokine IL-1β gene in the temporal cortex and increased the expression of the anti-inflammatory cytokine IL-1rn gene in all the brain structures examined. The possible explanation for why *B. longum* administration suppresses *Il1b* gene expression in the temporal cortex but not in the hippocampus may be related to the fact that the hippocampus is the brain structure with the highest density of IL-1 receptors [68]. Proinflammatory cytokines, especially IL-1b, are able to stimulate their own production by binding to their IL-1 receptors [69]. Therefore, when neuroinflammatory processes develop, abnormalities in the hippocampus are often more pronounced than in other areas of the brain.

Thus, *B. longum* limits neuroinflammatory processes that play an essential role in epileptogenesis [35,36,70,71]. The anti-inflammatory properties of *B. longum* have previously been shown in inflammatory bowel disease [72] and experimental colitis [73]. Furthermore, the administration of *B. longum* R0175 in combination with *Lactobacillus helveticus* R0052 in a model of neuroinflammation induced by the administration of bacterial lipopolysaccharide attenuated the increased gene expression of the proinflammatory cytokines IL-1β and TNF-α in hippocampal cells [74]. The suppression of the increased expression of some pro-inflammatory genes was also observed in a model of Alzheimer’s disease after the administration of *Bifidum* BGN4 and *B. longum* BORI [75]. The effect of *B. longum* on the development of neuroinflammatory processes in the TLE model has not yet been studied.

We also showed that *B. longum* enhances *Pparg* gene expression in the ventral hippocampus. PPARs are one of the key links in the interaction between the gut microbiota and brain [44]. They are involved in the regulation of lipid and glucose homeostasis, with PPARα (gene—*Ppara*) being more involved in fatty acid metabolism and the regulation of lipid levels; PPARβ/δ (gene—*Ppard*) is involved in fatty acid oxidation in muscles and the regulation of blood cholesterol levels; PPARγ (gene—*Pparg*) is associated with lipid biosynthesis, adipogenesis, and the regulation of energy balance [76]. PPAR ligands are short-chain fatty acids produced by intestinal microbiota [77]. *B. longum*, in combination with galacto-oligosaccharide, has been shown to increase the blood levels of short-chain fatty acids [78].

The involvement of PPARs in the regulation of pathological processes in epilepsy has been shown in a number of pharmacological studies using agonists of these receptors. For example, fenofibrate and bezafibrate, PPARα agonists, have been shown to have anticonvulsant properties in pentylenetetrazole and lithium–pilocarpine models of epilepsy [79,80]. The neuroprotective properties of PPARγ agonists have been identified in pentylenetetrazole-induced acute seizure [81] and in a chronic lithium–pilocarpine models [82]. In particular, the use of the PPARγ agonist rosiglitazone in a lithium–pilocarpine model of TLE was found to prevent cognitive impairment, increase antioxidant superoxide dismutase activity in brain cells [83], and reduce the inflammatory response of microglia [84]. Rosiglitazone also suppressed epileptiform discharges in an in vitro model of NMDA receptor-mediated epileptiform activity [85]. PPARγ has also been shown to mediate the anticonvulsant effects of a ketogenic diet, an effective treatment for pharmacoresistant epilepsy [86]. Taken together, these data suggest that the stimulatory effect of *B. longum* on *Pparg* gene expression that we identified can be considered as a neuroprotective effect.

Hippocampal sclerosis with neuronal loss and gliosis is characteristic of both patients with TLE [87] and experimental animals in TLE models [60,88]. In TLE models, reactive astrogliosis and microgliosis have also been reported in the piriform cortex [89], amygdala, and entorhinal cortex [90]. We observed similar morphological changes in this study. We hypothesized that the introduction of *B. longum* might attenuate these abnormalities. This assumption was based on the previous data on the ability of *B. longum* BORI combined with *B. bifidum* BGN4 to attenuate neuronal death in an experimental model of Alzheimer’s disease [75]. In addition, the ability of *B. longum* to inhibit microglial activity in the cerebellum of autistic-like rats was previously demonstrated [51].

In our experiments, *B. longum* reduced neuronal loss in the amygdala, but had almost no effect on the TLE-induced activation of microglia and astrocytes. Astrocytes and microglia are the major producers of both IL-1β and IL-1rn in the brain. The gene expression of these proteins was affected by the probiotic. The apparent contradiction in these results can be explained by the fact that microglia and astrocytes can be present in one of the alternative states, M1/M2 or A1/A2 (classical and alternative activation). M1 and A1 phenotypes produce pro-inflammatory proteins, while M2 and A2 produce anti-inflammatory proteins and neurotrophic factors [91,92,93]. The activation itself does not indicate the exact state of the micro- and astroglial cells (A1, M1 or A2, M2). Polarization regulation towards anti-inflammatory action may be an option for a therapeutic strategy to treat epilepsy [94]. Our results (*B. longum*-induced decrease in *Il1b* gene expression while increasing *Il1rn* gene expression along with microglia activation, especially in the temporal neocortex) suggest that *B. longum* may influence glial cell polarization. This assumption needs to be investigated in more detail. Thus, our findings indicate that an administration course of *B. longum* may have a protective effect on the lithium–pilocarpine model, and this probiotic may be recommended for further experimental and clinical studies as a possible regulator of epileptogenesis.

The attenuation of TLE-induced changes in the anxiety scores in rats by probiotic treatment was demonstrated in the present study using the OFT and SIT. This finding is consistent with the previous reports. A probiotic composition consisting of *L. helveticus* R0052 and *B. longum* R0175 reduced anxiety-like behavior in rats and psychological distress (as measured by the HSCL-90 scale) in volunteers [95]. In another experiment, the administration of *B. longum* 1714 to naturally anxious BALB/c mice reduced anxiety in the tail suspension test [96]. In studies on healthy humans, *B. longum* 1714 has been shown to reduce the severity of stress responses and to improve hippocampus-dependent visuospatial memory [48].

One of the reasons for the effect of B. longum on TLE-induced impairment of emotional behavior may be the attenuation of neuronal death and increased gene expression of the anti-inflammatory cytokine IL-1 receptor antagonist that we identified in the amygdala, which plays a key role in the regulation of anxiety levels [97]. The effects of *B. longum* on behavior may be mediated by increased cecal butyrate levels [98], changes in vagal activity [99], and increased synthesis of 5-hydroxytryptamine and brain-derived neurotrophic factor (BDNF) in the brain [98]. In addition, the effect of *B. longum* CCFM1077 on the levels of quinolinic acid, glutamic acid, and GABA in the brain of autistic-like rats was shown [51].

Overall, this study demonstrated some beneficial effects of the probiotic *B. longum* on a lithium–pilocarpine model of TLE. This study advances the understanding of the role of the gut microbiota in the pathogenesis of neuropsychiatric disorders, and *B. longum* appears promising for clinical trials in patients with epilepsy.

## 4. Materials and Methods

### 4.1. Animals

**Figure 11:**
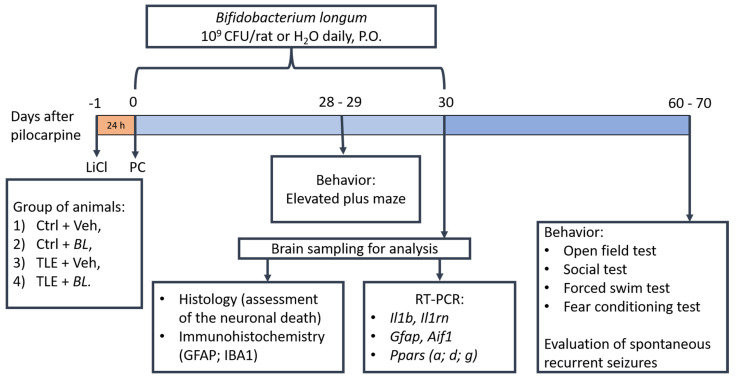
The experimental design.

Adult 7-week-old Wistar rats were used in this study. Because natural fluctuations in sex hormone levels during the ovarian cycle can affect the memory [100] and anxiety levels [101] of laboratory rodents, as well as interleukin-1 gene expression in their brains [102], the experiments were conducted on males only. The rats were housed four to six per cage at an ambient temperature of 22–25 °C under a 12 h day/night cycle with free access to food and water. All experiments were performed in accordance with the guidelines for handling laboratory animals of the Institute of Evolutionary Physiology and Biochemistry of the Russian Academy of Sciences (ethical approval number: 13-k-a, dated 15 February 2018). These guidelines are in accordance with the EU Directive 2010/63/EU on animal experimentation. Efforts were made to minimize animal suffering and to reduce the number of animals used. The design of the study is shown in Figure 11.

### 4.2. LiCl–Pilocarpine Model of TLE

The LiCl–pilocarpine rat model of TLE was established, as previously described [60,103,104]. Briefly, the rats were injected intraperitoneally (ip) with 127 mg/kg LiCl (Sigma-Aldrich, St. Louis, MO, USA) 24 h before pilocarpine injection. One hour before pilocarpine injection, (-)-scopolamine methyl bromide (1 mg/kg, ip; Sigma-Aldrich) was administered to block peripheral muscarinic receptors. Pilocarpine (Sigma-Aldrich) was administered at a total dose of 20–40 mg/kg (ip, 2 to 4 injections, 10 mg/kg, at 30 min intervals) to achieve stage 4 seizures on the Racine scale [105]. Diazepam (10 mg/kg, ip; Sigma-Aldrich) was administered 75 min after the onset of stage-4 seizures to terminate the seizures. Only rats that developed stage 4 seizures were included in the study. Control animals received the same drugs, except pilocarpine.

### 4.3. Probiotic Treatment

The control and experimental animals were randomly assigned to four groups: (1) TLE + *BL* (pilocarpine + probiotic *B. longum*, *n* = 16); (2) TLE + Veh (pilocarpine + water; *n* = 16); (3) Ctrl + *BL* (control + *B. longum*; *n* = 20); and (4) Ctrl + Veh (control + water; *n* = 18). *B. longum* (Custom Probiotics Inc., Glendale, CA, USA, 10^9^ CFU/rat, dissolved in 1 mL distilled water) or distilled water (1 mL) was administered once daily by oral gavage for 30 days.

### 4.4. Survival and Body Weight Dynamics

Survival and body weight dynamics were monitored daily for 1 month after pilocarpine administration. During the first 3 days, after pilocarpine injections, rats were administered a 5% glucose solution (2 mL, subcutaneously, daily) to improve survival outcomes. In addition, glucose injections were continued for several more days if the body weight dynamic was negative. Rats were also given wet chow during the first few days of the experiment.

### 4.5. mRNA Expression Analysis

The rats were decapitated 30 days after pilocarpine administration. The brain was rapidly removed and frozen at −80 °C. Areas of the dorsal (distance from bregma −2.4–−4.44) and ventral (distance from bregma −4.44–−5.28) parts of hippocampus, as well as the temporal cortex (distance from bregma −2.76–−4.68) and amygdala (distance from bregma −2.04–−3.0) were isolated according to a rat brain atlas [106] as previously described [104] and stored at −20 °C in an OTF5000 cryostat microtome (Bright Instruments, Luton, UK). Total RNA was extracted using ExtractRNA reagent (Evrogen, Moscow, Russia), according to the manufacturer’s instructions. RNA samples were treated with 1 unit of RQ1 DNase (Promega, Madison, WI, USA) for 15 min at 37 °C, followed by precipitation with 8 M LiCl (3 volumes of LiCl to 1 volume of RNA solution) and washed with 75% ethanol. RNA concentration and purity were assessed spectrophotometrically based on absorbance at 260 nm and an absorbance ratio 260/280, respectively, using a Nano Drop ™ Lite spectrophotometer (Thermo Fisher Scientific, Waltham, MA, USA).

cDNA was synthesized from 1 μg of total RNA with oligo-dT (0.5 μg per 1 μg RNA) and 9-mer random primers (0.25 μg per 1 μg RNA, DNA Sintez Ltd., Moscow, Russia) and 100 units of M-MLV reverse transcriptase (Evrogen, Moscow, Russia) in a total volume of 20 μL, according to the manufacturer’s protocol. All samples were 10-fold diluted before the PCR step. The primers and probes for *Gfap*, ionized calcium-binding adapter molecule 1 (*Aif1*), interleukin-1β (*Il1b*), interleukin-1 receptor antagonist (*Il1rn*), *Ppara*, *Ppard*, and *Pparg* (see Appendix C, Table A6) were purchased from DNA-Sintez Ltd. (Moscow, Russia). The primer and probe sequences were produced by Primer Blast software (https://www.ncbi.nlm.nih.gov/tools/primer-blast/, accessed on 25 March 2023) against cDNA sequences of *Aif1, Il1rn*, *Ppara*, *Ppard*, *Pparg* genes obtained from the National Center for Biotechnology Information (NCBI) Ref Seq database. In the cases of designing probes for previously described primer pairs, we used Primer3 Plus software version: 3.3.0 (Whitehead Institute for Biomedical Research, Cambridge, MA, USA), (https://primer3plus.com/cgi-bin/dev/primer3plus.cgi accessed on 25 March 2023), followed by nucleotide BLAST v2.10 specificity checking (https://blast.ncbi.nlm.nih.gov/Blast.cgi?PROGRAM=blastn&PAGE_TYPE=BlastSearch&LINK_LOC=blasthome accessed on 25 March 2023).

The qPCR was performed in a total volume of 6 μL with 0.8 μL of cDNA, 0.75 units of Taq M-polymerase (Alkor Bio, St. Petersburg, Russia), 3.5 mM MgCl_2_, and specific primers and probes (Table A6). All samples were analyzed in tetraplicates. The qPCR was performed on a C1000 Touch thermal cycler in combination with a CFX384 Touch™ real-time PCR detection system (Bio-Rad, Hercules, CA, USA).

The relative expression of the genes of interest was calculated using the 2^−ΔΔCt^ method, normalized to the geometric mean for the three most stable reference genes in the brain region analyzed, and evaluated as described elsewhere [107]. The expressions of 9 reference genes (*Actb*, *Gapdh*, *B2m*, *Rpl13a*, *Sdha*, *Ppia*, *Hprt1*, *Pgk1*, *Ywhaz*) were tested using 3 triplex assays previously described [108]. Reference genes for the data normalization were selected based on a comprehensive ranking, obtained using the Ref Finder^®^ online tool (https://www.heartcure.com.au/reffinder/ accessed on 25 March 2023), which incorporates GeNorm, Norm Finder, Best Keeper, and comparative delta CT algorithms. For qPCR data normalization, the following reference genes were selected: *Hprt1*, *Pgk1*, *Ywhaz* in the dorsal hippocampus; *Actb*, *Sdha*, *Hprt1* in the ventral hippocampus; *Rpl13*, *Sdha*, *Ywhaz* in the temporal cortex; and *Actb*, *Rpl13*, *Pgk1* in amygdala.

### 4.6. Histological and Immunohistochemical Procedures

The separate group of animals were subjected to the same experimental protocols and sacrificed on day 30 post-SE to assess the histological changes in the temporal lobe.

After decapitation, the brains were rapidly removed and fixed in 10% formalin for 72 h. Paraffin blocks were then prepared and serial sections were created. At least three 3 μm sections between bregma −2.5 and −4.5 mm were obtained for analysis.

For the histological analysis, sections were Nissl-stained and the total neuronal densities in the hippocampal hilus, CA1 region, as well as amygdala and temporal neocortex were counted using ImageJ software, Version 1.53t. The neuronal density (the number of neurons per square millimeter) was manually counted throughout the CA1 region in three non-overlapping areas (×400): from CA2 (characteristic Ammon’s horn cell thickening) to the subiculum (characteristic Ammon’s horn cell dispersion). In the hilus, the neuronal density was counted between the subgranular layer of the dentate gyrus and the beginning of the CA3 region in two non-overlapping areas (×200). In the temporal neocortex and amygdala, two non-overlapping areas (×200) were used for counting in all cases. The density values in all regions were averaged for each animal and then used for the analysis.

Rabbit anti-glial fibrillary acidic protein (GFAP) primary antibody (1:10,000, FineTest, Cat. No. fNab03428) was used for the immunohistochemical identification of astroglia. Rabbit anti-ionized calcium-binding adapter molecule 1 (IBA1) antibody (1:10,000, FineTest, Palm Coast, FL, USA, Cat. No. fNab04096) was used for the analysis of the expression of microglia. UnoVue Mouse/Rabbit HRP Kit (Diagnostics BioSystems, Pleasanton, CA, USA, Cat. No. UMR1000PD) with secondary antibodies and DAB as chromogen was used for visualization. Mayer’s hematoxylin was used for counterstaining. Cell bodies were counted manually in dorsal CA1, the hilus, and temporal neocortex in the same number of areas, as described above, for histology.

### 4.7. Behavioral Tests

For this study, we chose tests that have previously shown behavioral abnormalities in rats in the TLE model [59,60]. Anxiety tests were also used because of the ability of *B. longum* to normalize anxiety-like behavior has been previously identified [109]. In total, five behavioral tests were conducted (Figure 11). The order in which the tests were performed was the same for all groups. No more than one test was performed each day. The experimenters evaluating the behavior were not informed about the distribution of the rats into groups.

#### 4.7.1. Open Field Test (OFT)

The diameter of the circular open field was 1 m with a wall height of 30 cm. Illumination was 8 lux. The rat was placed in the center of the arena for 5 min and the behavior was videotaped. The recordings were analyzed offline using “Tracking”, Version 3.2 and “Field 4” Version 4 software (Institute of Experimental Medicine, St. Petersburg, Russia). We evaluated the length of the track and the characteristics of locomotor activity in different areas of the field [110]. To assess the level of anxiety, we analyzed the time spent in the center of the arena (1/2 its diameter). In addition, we considered the timing and number of behavioral patterns: climbing (as an indicator of motor and exploratory activity), self-grooming, rearing, and freezing (as indicators of anxiety level) [110].

#### 4.7.2. Elevated Plus Maze (EPM)

The maze consisted of a central platform (10 × 10 cm) with two open and two closed arms (each 50 × 10 cm) at a height of 40 cm above the floor. The closed arms had 30 cm walls and an opaque cover. Illumination was 1 lux in the closed arms and 40 lux in the open arms. The rat was placed in the center of the maze facing one of the open arms. The test lasted 5 min. The behavior was recorded by webcam. The time spent in the open and closed arms was used to assess the level of anxiety [111].

#### 4.7.3. Social Interaction Test (SIT)

The SIT was used to assess social behavior [112]. The rats were placed in a Plexiglas cage (60 × 30 cm, height = 40 cm) for 30 min prior to the test to reduce anxiety and exploratory behaviors related to the novel environment. An unfamiliar adult intact male Wistar rat was then placed in the same cage for 5 min. The following patterns were recorded and measured: communication (sniffing and grooming of the intruder’s body), aggression, defensive behavior, and noncommunicative behavior. The resident’s self-grooming behavior was analyzed as a measure of the animal’s anxiety.

#### 4.7.4. Forced *Swim Test (FST)*

The FST was used to analyze depressive-like behavior [113]. We performed two sessions with animals placed in a cylinder (diameter 40 cm, height 70 cm) containing 25 °C water. During the first 15 min adaptation session, no behavior was recorded. The test was repeated 24 h later in a 5 min session. The duration of immobilization was estimated.

#### 4.7.5. Fear Conditioning Test (FCT)

The FCT was used to investigate memory associated with fear and was conducted as previously described [59]. Two Plexiglas cages were used for this test. Cage A (45 × 30 cm, height = 20 cm) had an electrically conductive floor. Cage B was larger (60 × 30 cm, height = 40 cm) and had no conductive floor. On day zero (habituation day), the rat was placed in cage A for 5 min for habituation. On the first day (conditioning day), the rat was placed in the same cage A. After 2 min of habituation (step 1–1), an 80 dB tone (conditioning signal) was delivered for 22 s, accompanied by a mild foot shock (0.6 mA current on the floor, an aversive stimulus) during the last 2 s (step 1–2). After a 2 min break (step 1–3), step 1–2 was repeated (step 1–4), and no tone or aversive stimulus was delivered during the last 1 min of the test (step 1–5). On the second day (test day), the rat was placed in cage A for 3 min (step 2–1) without an electric current or tone to assess the fear responses (contextual conditioning test). The rat was then moved to a larger cage with a new odor (vanillin drops on the floor) and a new interior design (pictures of geometric figures on the walls). The new context was used to assess the associations only with the sound and not with the cage (conditioning test). No stimuli were presented for the first 3 min (step 2–2). An 80 dB tone was then presented for 3 min to assess the fear response to the tone associated with the current (step 2–3). During the last 1 min of the test, the rat was not exposed to any stimuli (phase 2–4). During each phase, the total freezing time was measured and counted as a reflection of the fear response and fear-related memory. The percentage of freezing in all phases of the test was then calculated.

### 4.8. Evaluation of SRSs

To assess the frequency of SRSs, the rats were housed in individual cages with clear walls, with free access to food and water. The rats were continuously videotaped for 48 h.

### 4.9. Statistical Analysis

Statistical analysis was performed using IBM SPSS Statistics 20 (IBM, Armonk, NY, USA) and Graph Pad Prism 8 software (Graph Pad Software, San Diego, CA, USA). Outliers were detected using Dixon’s Q-test. Normality of distribution was tested by the Kolmogorov–Smirnov test. The effects of probiotic treatment were performed using two-way ANOVA (for normally distributed data) or Kruskal–Wallis H test (non-normal distribution). Post hoc analysis was performed using Sidak’s or Dunn’s multiple comparison tests, respectively. When one-way ANOVA was used, the Welch test with Games–Howell post hoc test was used for significant differences in the variance between groups. Survival analysis with the log-rank Mantel–Cox test calculation was also used. Differences were considered significant when *p* ≤ 0.05. The data are presented as mean ± standard error of the mean (normal distribution) or median and interquartile range (non-normal distribution).

## Data Availability

The data presented in this study are available on request from the corresponding author.

## Appendix B. Results of Statistical Analysis

**Table A1:** Complete results of the statistical analysis for the data shown in Figure 4.

Structures	Factors	*Ppara*	*Ppard*	*Pparg*
Dorsal hippocampus	Seizures	F_1,25_ = 11.9, *p* = 0.002	F_1,24_ = 1.6, *p* = 0.22	F_1,24_ = 0.0, *p* = 0.99
	*B. longum*	F_1,25_ = 1.3, *p* = 0.26	F_1,24_ = 1.9, *p* = 0.18	F_1,24_ = 1.8, *p* = 0.19
	Interaction	F_1,25_ = 1.4, *p* = 0.25	F_1,24_ = 0.03, *p* = 0.86	F_1,24_ = 0.08, *p* = 0.73
Ventral hippocampus	Seizures	F_1,22_ = 0.5, *p* = 0.49	F_1,23_ = 4.3, *p* = 0.05	F_1,19_ = 6.2, *p* = 0.020
	*B. longum*	F_1,22_ = 0.04, *p* = 0.85	F_1,23_ = 0.0, *p* = 0.94	F_1,19_ = 13.3, *p* = 0.002
	Interaction	F_1,22_ = 0.6, *p* = 0.45	F_1,23_ = 0.0, *p* = 0.95	F_1,19_ = 1.7, *p* = 0.21
Temporal cortex	Seizures	F_1,26_ = 0.7, *p* = 0.41	F_1,27_ = 0.6, *p* = 0.45	F_1,25_ = 0.1, *p* = 0.77
	*B. longum*	F_1,26_ = 0.06, *p* = 0.81	F_1,27_ = 0.03, *p* = 0.86	F_1,25_ = 1.8, *p* = 0.20
	Interaction	F_1,26_ = 2.1, *p* = 0.16	F_1,27_ = 0.35, *p* = 0.56	F_1,25_ = 0.5, *p* = 0.48
Amygdala	Seizures	F_1,25_ = 2.1, *p* = 0.16	F_1,24_ = 0.1, *p* = 0.70	F_1,25_ = 0.9, *p* = 0.36
	*B. longum*	F_1,25_ = 0.06, *p* = 0.82	F_1,24_ = 0.8, *p* = 0.39	F_1,25_ = 2.9, *p* = 0.10
	Interaction	F_1,25_ = 0.1, *p* = 0.79	F_1,24_ = 3.0, *p* = 0.10	F_1,25_ = 0.8, *p* = 0.38

**Table A2:** Full results of statistical analysis of behavioral indicators in the OFT.

Indicators	Factors		
	TLE	*B. longum*	Interaction
Climbing time	F_1,65_ = 16.30; *p* < 0.001	F_1,65_ = 1.33; *p* = 0.25	F_1,65_ = 0.002; *p* = 0.96
Grooming time	F_1,61_ = 4.45; *p* = 0.04	F_1,61_ = 0.15; *p* = 0.69	F_1,61_ = 0.12; *p* = 0.73
Rearing, number *	H = 5.2; *p* = 0.16		
Rearing, time (s) *	H = 2.6; *p* = 0.46		
Freezing, time (s) *	H = 0.9; *p* = 0.82		

* Data are distributed abnormally.

**Table A3:** Full results of statistical analysis for behavioral indicators in EPM and FST.

	Factors		
	TLE	*B. longum*	Interaction
EPM, time in closed arms	F_1,54_ = 0.11; *p* = 0.74	F_1,54_ = 10.09; *p* = 0.30	F_1,54_ = 3.24; *p* = 0.08
EPM, time in open arms	F_1,54_ = 10.77; *p* = 0.002	F_1,54_ = 2.84; *p* = 0.10	F_1,54_ = 1.48; *p* = 0.23
EPM, ratio time in open arms/total time in arms *	H = 14.1; *p* = 0.003.		
FST, time of immobilization, s	F_1,29_ = 6.46; *p* = 0.02	F_1,29_ = 0.22; *p* = 0.64	F_1,29_ = 0.06; *p* = 0.81

* Data are distributed abnormally.

**Table A4:** Full results of statistical analysis for behavioral indicators in FCT.

	Factors		
	TLE	*B. longum*	Interaction
Familiar cage	F_1,32_ = 23.1; *p* < 0.001	F_1,32_ = 2.7; *p* = 0.11	F_1,32_ = 0.9; *p* = 0.34
Novel cage, test 1, time of freezing during adaptation	F_1,32_ = 4.1; *p* = 0.051	F_1,32_ = 0.9; *p* = 0.35	F_1,32_ = 1.2; *p* = 0.28
Novel cage, test 2, time of freezing during the conditioned stimulus (sound)	F_1,32_ = 19.5; *p* < 0.001	F_1,32_ = 0.3; *p* = 0.59	F_1,32_ = 0.2; *p* = 0.89
Novel cage, test 3, time of freezing after the conditioned stimulus	F_1,33_ = 1.9; *p* = 0.17	F_1,33_ = 0.01; *p* = 0.93	F_1,33_ = 0.01; *p* = 0.96

**Table A5:** Full results of statistical analysis for behavioral indicators in SIT.

	Factors		
	TLE	*B. longum*	Interaction
Time of communication, s	F_1,34_ = 6.80; *p* = 0.01	F_1,34_ = 1.42; *p* = 0.24	F_1,34_ = 0.18; *p* = 0.68
Time of self-grooming, s	F_1,34_ = 13.8; *p* = 0.001	F_1,34_ = 1.35; *p* = 0.25	F_1,34_ = 2.19; *p* = 0.15
Average time of one self-grooming, s	F_1,32_ = 20.24; *p* < 0.001	F_1,32_ = 1.61; *p* = 0.21	F_1,32_ = 4.33; *p* = 0.045
Number of self-grooming episodes	F_1,35_ = 0.98; *p* = 0.32	F_1,35_ = 0.10; *p* = 0.75	F_1,35_ = 0.27; *p* = 0.61
Time of aggressive behavior, s *	H = 4.05; *p* = 0.26		

* Data are distributed abnormally.

## Appendix C

**Table A6:** Nucleotide sequences.

Gene Symbol RefSeq Accession Number	Nucleotide Sequences (Forward, Reverse, TaqMan Probe)	Reference
*Ppara* NM_013196.2	AATCCACGAAGCCTACCTGA GTCTTCTCAGCCATGCACAA FAM-AGGCCCGGGTCATACTCGCAGGAA-BHQ1	[114] (primers) This work (probe)
*Ppard* NM_013141.2	CAAACCCACGGTAAAGGCGG TGGCTGTTCCATGACTGACC HEX-CCAGGCCTGCAGGCGCCACGCCA-BHQ2	This work
*Pparg* NM_013124.3	CCTGAAGCTCCAAGAATACC GATGCTTTATCCCCACAGAC HEX-CCCTCATGGCCATCGAGTGCC-BHQ2	[115] (primers) This work (probe)
*Gfap* NM_017009.2	TGGCCACCAGTAACATGCAA CAGTTGGCGGCGATAGTCAT HEX-CGGTCCAAGTTTGCAGACCTCACAG-BHQ2	[116] (primers) [60] (probe)
*Il1b* NM_031512	CACCTCTCAAGCAGAGCACAG GGGTTCCATGGTGAAGTCAAC FAM-TGTCCCGACCATTGCTGTTTCCTAG-BHQ1	[117]
*Aif1* NM_017196.3	CAACACACTGCAGCCTCATC AAGCTTTTCCTCCCTGCAAA Cy5-CCCCACCTAAGGCCACCAGCGTCTGA-BHQ3	This work
*Il1rn* NM_022194.2	GGGGACCTTACAGTCACCTAAT GGTTAGTATCCCAGATTCTGAAGG ROX-AGTCAGCTGGCCACCCTGCTGGGA-BHQ2	This work
*Actb* NM_031144	TGTCACCAACTGGGACGATA GGGGTGTTGAAGGTCTCAAA FAM-CGTGTGGCCCCTGAGGAGCAC-BHQ1	[118] (primers) [108] (probe)
*Gapdh* NM_017008	TGCACCACCAACTGCTTAG GGATGCAGGGATGATGTTC R6G-ATCACGCCACAGCTTTCCAGAGGG-BHQ2	[119]
*B2m* NM_012512	TGCCATTCAGAAAACTCCCC GAGGAAGTTGGGCTTCCCATT ROX-ATTCAAGTGTACTCTCGCCATCCACCG-BHQ1	[120]
*Rpl13a* NM_173340	GGATCCCTCCACCCTATGACA CTGGTACTTCCACCCGACCTC FAM-CTGCCCTCAAGGTTGTGCGGCT-BHQ1	[121] (primers) [108] (probe)
*Sdha* NM_130428	AGACGTTTGACAGGGGAATG TCATCAATCCGCACCTTGTA R6G-ACCTGGTGGAGACGCTGGAGCT-BHQ2	[122] (primers) [108] (probe)
*Ppia* NM_017101	AGGATTCATGTGCCAGGGTG CTCAGTCTTGGCAGTGCAGA ROX-CACGCCATAATGGCACTGGTGGCA-BHQ1	[123]
*Hprt1* NM_012583	TCCTCAGACCGCTTTTCCCGC TCATCATCACTAATCACGACGCTGG FAM-CCGACCGGTTCTGTCATGTCGACCCT-BHQ1	[124] (primers) [108] (probe)
*Pgk1* NM_053291	ATGCAAAGACTGGCCAAGCTAC AGCCACAGCCTCAGCATATTTC R6G-TGCTGGCTGGATGGGCTTGGA-BHQ2	[125] (primers) [108] (probe)
*Ywhaz* NM_013011	GATGAAGCCATTGCTGAACTTG GTCTCCTTGGGTATCCGATGTC ROX-TGAAGAGTCGTACAAAGACAGCACGC-BHQ1	[125] (primers) [108] (probe)

## References

[1] Fiest K.M., Sauro K.M., Wiebe S., Patten S.B., Kwon C.-S., Dykeman J., Pringsheim T., Lorenzetti D.L., Jetté N. (2017). Prevalence and incidence of epilepsy. Neurology.

[2] Al Sufiani F., Ang L.C. (2012). Neuropathology of Temporal Lobe Epilepsy. Epilepsy Res. Treat..

[3] Vrinda M., Arun S., Srikumar B.N., Kutty B.M., Shankaranarayana Rao B.S. (2019). Temporal lobe epilepsy-induced neurodegeneration and cognitive deficits: Implications for aging. J. Chem. Neuroanat..

[4] Johnson A.M., Sugo E., Barreto D., Hiew C.-C., Lawson J.A., Connolly A.M., Somerville E., Hasic E., Bye A.M., Cunningham A.M. (2016). The Severity of Gliosis in Hippocampal Sclerosis Correlates with Pre-Operative Seizure Burden and Outcome after Temporal Lobectomy. Mol. Neurobiol..

[5] Tramoni-Negre E., Lambert I., Bartolomei F., Felician O. (2017). Long-term memory deficits in temporal lobe epilepsy. Rev. Neurol..

[6] Rini J.F., Ochoa J. (2020). Behavioral implications of temporal lobe epilepsy on social contingency. Epilepsy Behav..

[7] Vinti V., Dell’Isola G.B., Tascini G., Mencaroni E., Cara G.D., Striano P., Verrotti A. (2021). Temporal Lobe Epilepsy and Psychiatric Comorbidity. Front. Neurol..

[8] Fattorusso A., Matricardi S., Mencaroni E., Dell’Isola G.B., Di Cara G., Striano P., Verrotti A. (2021). The Pharmacoresistant Epilepsy: An Overview on Existant and New Emerging Therapies. Front. Neurol..

[9] Walia K.S., Khan E.A., Ko D.H., Raza S.S., Khan Y.N. (2004). Side Effects of Antiepileptics—A Review. Pain Pract..

[10] Löscher W. (2020). The holy grail of epilepsy prevention: Preclinical approaches to antiepileptogenic treatments. Neuropharmacology.

[11] Parashar A., Udayabanu M. (2017). Gut microbiota: Implications in Parkinson’s disease. Parkinsonism Relat. Disord..

[12] Mangiola F. (2016). Gut microbiota in autism and mood disorders. World J. Gastroenterol..

[13] Limbana T., Khan F., Eskander N. (2020). Gut Microbiome and Depression: How Microbes Affect the Way We Think. Cureus.

[14] Mörkl S., Butler M.I., Holl A., Cryan J.F., Dinan T.G. (2020). Probiotics and the Microbiota-Gut-Brain Axis: Focus on Psychiatry. Curr. Nutr. Rep..

[15] Shaikh M.F., Lee C.Y., Chen W.N., Shaikh F.A. (2020). The Gut-Brain-Axis on the Manifestation of Depressive Symptoms in Epilepsy: An Evidence-Driven Hypothesis. Front. Pharmacol..

[16] Iannone L.F., Preda A., Blottière H.M., Clarke G., Albani D., Belcastro V., Carotenuto M., Cattaneo A., Citraro R., Ferraris C. (2019). Microbiota-gut brain axis involvement in neuropsychiatric disorders. Expert Rev. Neurother..

[17] Yue Q., Cai M., Xiao B., Zhan Q., Zeng C. (2022). The Microbiota–Gut–Brain Axis and Epilepsy. Cell. Mol. Neurobiol..

[18] De Caro C., Iannone L.F., Citraro R., Striano P., De Sarro G., Constanti A., Cryan J.F., Russo E. (2019). Can we ‘seize’ the gut microbiota to treat epilepsy?. Neurosci. Biobehav. Rev..

[19] Chen C.-H., Lin C.-L., Kao C.-H. (2015). Irritable Bowel Syndrome Increases the Risk of Epilepsy. Medicine.

[20] Pittayanon R., Lau J.T., Yuan Y., Leontiadis G.I., Tse F., Surette M., Moayyedi P. (2019). Gut Microbiota in Patients with Irritable Bowel Syndrome—A Systematic Review. Gastroenterology.

[21] Holmes M., Flaminio Z., Vardhan M., Xu F., Li X., Devinsky O., Saxena D. (2020). Cross talk between drug-resistant epilepsy and the gut microbiome. Epilepsia.

[22] Gong X., Liu X., Chen C., Lin J., Li A., Guo K., An D., Zhou D., Hong Z. (2020). Alteration of Gut Microbiota in Patients with Epilepsy and the Potential Index as a Biomarker. Front. Microbiol..

[23] Peng A., Qiu X., Lai W., Li W., Zhang L., Zhu X., He S., Duan J., Chen L. (2018). Altered composition of the gut microbiome in patients with drug-resistant epilepsy. Epilepsy Res..

[24] Xie G., Zhou Q., Qiu C.-Z., Dai W.-K., Wang H.-P., Li Y.-H., Liao J.-X., Lu X.-G., Lin S.-F., Ye J.-H. (2017). Ketogenic diet poses a significant effect on imbalanced gut microbiota in infants with refractory epilepsy. World J. Gastroenterol..

[25] Huang C., Li Y., Feng X., Li D., Li X., Ouyang Q., Dai W., Wu G., Zhou Q., Wang P. (2019). Distinct Gut Microbiota Composition and Functional Category in Children with Cerebral Palsy and Epilepsy. Front. Pediatr..

[26] Lee K., Kim N., Shim J.O., Kim G.-H. (2020). Gut Bacterial Dysbiosis in Children with Intractable Epilepsy. J. Clin. Med..

[27] Şafak B., Altunan B., Topçu B., Eren Topkaya A. (2020). The gut microbiome in epilepsy. Microb. Pathog..

[28] Gómez-Eguílaz M., Ramón-Trapero J.L., Pérez-Martínez L., Blanco J.R. (2018). The beneficial effect of probiotics as a supplementary treatment in drug-resistant epilepsy: A pilot study. Benef. Microbes.

[29] Socała K., Doboszewska U., Szopa A., Serefko A., Włodarczyk M., Zielińska A., Poleszak E., Fichna J., Wlaź P. (2021). The role of microbiota-gut-brain axis in neuropsychiatric and neurological disorders. Pharmacol. Res..

[30] Hingray C., McGonigal A., Kotwas I., Micoulaud-Franchi J.-A. (2019). The Relationship between Epilepsy and Anxiety Disorders. Curr. Psychiatry Rep..

[31] Bagheri S., Heydari A., Alinaghipour A., Salami M. (2019). Effect of probiotic supplementation on seizure activity and cognitive performance in PTZ-induced chemical kindling. Epilepsy Behav..

[32] Tahmasebi S., Oryan S., Mohajerani H.R., Akbari N., Palizvan M.R. (2020). Probiotics and *Nigella sativa* extract supplementation improved behavioral and electrophysiological effects of PTZ-induced chemical kindling in rats. Epilepsy Behav..

[33] Aygun H., Akin A.T., Kızılaslan N., Sumbul O., Karabulut D. (2022). Probiotic supplementation alleviates absence seizures and anxiety- and depression-like behavior in WAG/Rij rat by increasing neurotrophic factors and decreasing proinflammatory cytokines. Epilepsy Behav..

[34] Yu L.W., Agirman G., Hsiao E.Y. (2022). The Gut Microbiome as a Regulator of the Neuroimmune Landscape. Annu. Rev. Immunol..

[35] Pracucci E., Pillai V., Lamers D., Parra R., Landi S. (2021). Neuroinflammation: A Signature or a Cause of Epilepsy?. Int. J. Mol. Sci..

[36] Rana A., Musto A.E. (2018). The role of inflammation in the development of epilepsy. J. Neuroinflamm..

[37] Mendiola A.S., Cardona A.E. (2018). The IL-1β phenomena in neuroinflammatory diseases. J. Neural Transm..

[38] Soltani Khaboushan A., Yazdanpanah N., Rezaei N. (2022). Neuroinflammation and Proinflammatory Cytokines in Epileptogenesis. Mol. Neurobiol..

[39] Hutchinson P.J., O’Connell M.T., Rothwell N.J., Hopkins S.J., Nortje J., Carpenter K.L.H., Timofeev I., Al-Rawi P.G., Menon D.K., Pickard J.D. (2007). Inflammation in Human Brain Injury: Intracerebral Concentrations of IL-1 α, IL-1 β, and Their Endogenous Inhibitor IL-1ra. J. Neurotrauma.

[40] Vezzani A., Moneta D., Richichi C., Aliprandi M., Burrows S.J., Ravizza T., Perego C., De Simoni M.G. (2002). Functional Role of Inflammatory Cytokines and Antiinflammatory Molecules in Seizures and Epileptogenesis. Epilepsia.

[41] Frank M.G., Fonken L.K., Watkins L.R., Maier S.F., Lowry C.A. (2019). Could Probiotics Be Used to Mitigate Neuroinflammation?. ACS Chem. Neurosci..

[42] Zolezzi J.M., Santos M.J., Bastías-Candia S., Pinto C., Godoy J.A., Inestrosa N.C. (2017). PPARs in the central nervous system: Roles in neurodegeneration and neuroinflammation. Biol. Rev..

[43] Hong F., Pan S., Guo Y., Xu P., Zhai Y. (2019). PPARs as Nuclear Receptors for Nutrient and Energy Metabolism. Molecules.

[44] Zubareva O.E., Melik-Kasumov T.B. (2021). The Gut–Brain Axis and Peroxisome Proliferator-Activated Receptors in the Regulation of Epileptogenesis. J. Evol. Biochem. Physiol..

[45] Di Paola M., Bonechi E., Provensi G., Costa A., Clarke G., Ballerini C., De Filippo C., Passani M.B. (2018). Oleoylethanolamide treatment affects gut microbiota composition and the expression of intestinal cytokines in Peyer’s patches of mice. Sci. Rep..

[46] Kim S., Park S., Choi T.G., Kim S.S. (2022). Role of Short Chain Fatty Acids in Epilepsy and Potential Benefits of Probiotics and Prebiotics: Targeting “Health” of Epileptic Patients. Nutrients.

[47] Wang X., Ma R., Liu X., Zhang Y. (2022). Effects of long-term supplementation of probiotics on cognitive function and emotion in temporal lobe epilepsy. Front. Neurol..

[48] Allen A.P., Hutch W., Borre Y.E., Kennedy P.J., Temko A., Boylan G., Murphy E., Cryan J.F., Dinan T.G., Clarke G. (2016). *Bifidobacterium longum* 1714 as a translational psychobiotic: Modulation of stress, electrophysiology and neurocognition in healthy volunteers. Transl. Psychiatry.

[49] Curia G., Longo D., Biagini G., Jones R.S.G., Avoli M. (2008). The pilocarpine model of temporal lobe epilepsy. J. Neurosci. Methods.

[50] Plata A., Lebedeva A., Denisov P., Nosova O., Postnikova T.Y., Pimashkin A., Brazhe A., Zaitsev A.V., Rusakov D.A., Semyanov A. (2018). Astrocytic Atrophy Following Status Epilepticus Parallels Reduced Ca2+ Activity and Impaired Synaptic Plasticity in the Rat Hippocampus. Front. Mol. Neurosci..

[51] Kong Q., Chen Q., Mao X., Wang G., Zhao J., Zhang H., Chen W. (2022). *Bifidobacterium longum* CCFM1077 Ameliorated Neurotransmitter Disorder and Neuroinflammation Closely Linked to Regulation in the Kynurenine Pathway of Autistic-like Rats. Nutrients.

[52] Chakir A., Fabene P.F., Ouazzani R., Bentivoglio M. (2006). Drug resistance and hippocampal damage after delayed treatment of pilocarpine-induced epilepsy in the rat. Brain Res. Bull..

[53] Postnikova T.Y., Diespirov G.P., Amakhin D.V., Vylekzhanina E.N., Soboleva E.B., Zaitsev A.V. (2021). Impairments of Long-Term Synaptic Plasticity in the Hippocampus of Young Rats during the Latent Phase of the Lithium-Pilocarpine Model of Temporal Lobe Epilepsy. Int. J. Mol. Sci..

[54] Borges K. (2003). Neuronal and glial pathological changes during epileptogenesis in the mouse pilocarpine model. Exp. Neurol..

[55] Borges K., McDermott D., Irier H., Smith Y., Dingledine R. (2006). Degeneration and proliferation of astrocytes in the mouse dentate gyrus after pilocarpine-induced status epilepticus. Exp. Neurol..

[56] Eid T., Lee T.S.W., Patrylo P., Zaveri H.P. (2019). Astrocytes and Glutamine Synthetase in Epileptogenesis. J. Neurosci. Res..

[57] Victor T.R., Tsirka S.E. (2020). Microglial contributions to aberrant neurogenesis and pathophysiology of epilepsy. Neuroimmunol. Neuroinflamm..

[58] Zaitsev А.V., Amakhin D.V., Dyomina A.V., Zakharova M.V., Ergina J.L., Postnikova T.Y., Diespirov G.P., Magazanik L.G. (2021). Synaptic Dysfunction in Epilepsy. J. Evol. Biochem. Physiol..

[59] Smolensky I.V., Zubareva O.E., Kalemenev S.V., Lavrentyeva V.V., Dyomina A.V., Karepanov A.A., Zaitsev A.V. (2019). Impairments in cognitive functions and emotional and social behaviors in a rat lithium-pilocarpine model of temporal lobe epilepsy. Behav. Brain Res..

[60] Dyomina A.V., Zubareva O.E., Smolensky I.V., Vasilev D.S., Zakharova M.V., Kovalenko A.A., Schwarz A.P., Ischenko A.M., Zaitsev A.V. (2020). Anakinra Reduces Epileptogenesis, Provides Neuroprotection, and Attenuates Behavioral Impairments in Rats in the Lithium–Pilocarpine Model of Epilepsy. Pharmaceuticals.

[61] Kalueff A.V., Stewart A.M., Song C., Berridge K.C., Graybiel A.M., Fentress J.C. (2016). Neurobiology of rodent self-grooming and its value for translational neuroscience. Nat. Rev. Neurosci..

[62] Ma T., Jin H., Kwok L.-Y., Sun Z., Liong M.-T., Zhang H. (2021). Probiotic consumption relieved human stress and anxiety symptoms possibly via modulating the neuroactive potential of the gut microbiota. Neurobiol. Stress.

[63] Wang H., Lee I.-S., Braun C., Enck P. (2016). Effect of Probiotics on Central Nervous System Functions in Animals and Humans: A Systematic Review. J. Neurogastroenterol. Motil..

[64] Garrido D., Ruiz-Moyano S., Jimenez-Espinoza R., Eom H.-J., Block D.E., Mills D.A. (2013). Utilization of galactooligosaccharides by *Bifidobacterium longum* subsp. infantis isolates. Food Microbiol..

[65] Li Y., Wang S., Sun Y., Zheng H., Tang Y., Gao X., Song C., Liu J., Long Y., Liu L. (2020). Apple polysaccharide could promote the growth of *Bifidobacterium longum*. Int. J. Biol. Macromol..

[66] Chaskiel L., Bristow A.D., Bluthé R.-M., Dantzer R., Blomqvist A., Konsman J.P. (2019). Interleukin-1 reduces food intake and body weight in rat by acting in the arcuate hypothalamus. Brain. Behav. Immun..

[67] Zubareva O.E., Krasnova I.N., Abdurasulova I.N., Bluthe R.-M., Dantzer R., Klimenko V.M. (2001). Effects of serotonin synthesis blockade on interleukin-1β action in the brain of rats. Brain Res..

[68] Takao T., Tracey D.E., Mark Mitchell W., de Souza E.B. (1990). Interleukin-1 receptors in mouse brain: Characterization and neuronal localization. Endocrinology.

[69] Basu A., Krady J.K., Levison S.W. (2004). Interleukin-1: A master regulator of neuroinflammation. J. Neurosci. Res..

[70] Shimada T., Takemiya T., Sugiura H., Yamagata K. (2014). Role of Inflammatory Mediators in the Pathogenesis of Epilepsy. Mediators Inflamm..

[71] Vezzani A., Balosso S., Ravizza T. (2008). The role of cytokines in the pathophysiology of epilepsy. Brain. Behav. Immun..

[72] Underwood M.A., Arriola J., Gerber C.W., Kaveti A., Kalanetra K.M., Kananurak A., Bevins C.L., Mills D.A., Dvorak B. (2014). *Bifidobacterium longum* subsp. infantis in experimental necrotizing enterocolitis: Alterations in inflammation, innate immune response, and the microbiota. Pediatr. Res..

[73] Abrantes F.A., Nascimento B.B., Andrade M.E.R., de Barros P.A.V., Cartelle C.T., Martins F.S., Nicoli J.R., Arantes R.M.E., Generoso S.V., Fernandes S.O.A. (2020). Treatment with *Bifidobacterium longum* 5 ^1A^ attenuates intestinal damage and inflammatory response in experimental colitis. Benef. Microbes.

[74] Mohammadi G., Dargahi L., Peymani A., Mirzanejad Y., Alizadeh S.A., Naserpour T., Nassiri-Asl M. (2019). The Effects of Probiotic Formulation Pretreatment (*Lactobacillus helveticus* R0052 and *Bifidobacterium longum* R0175) on a Lipopolysaccharide Rat Model. J. Am. Coll. Nutr..

[75] Kim H., Kim S., Park S., Park G., Shin H., Park M.S., Kim J. (2021). Administration of *Bifidobacterium bifidum* BGN4 and *Bifidobacterium longum* BORI Improves Cognitive and Memory Function in the Mouse Model of Alzheimer’s Disease. Front. Aging Neurosci..

[76] Grygiel-Górniak B. (2014). Peroxisome proliferator-activated receptors and their ligands: Nutritional and clinical implications—A review. Nutr. J..

[77] Gervois P., Torra I.P., Fruchart J.-C., Staels B. (2000). Regulation of Lipid and Lipoprotein Metabolism by PPAR Activators. Clin. Chem. Lab. Med..

[78] Kim D., Lee K.R., Kim N.R., Park S.-J., Lee M., Kim O.-K. (2021). Combination of *Bifidobacterium longum* and Galacto-Oligosaccharide Protects the Skin from Photoaging. J. Med. Food.

[79] Saha L., Bhandari S., Bhatia A., Banerjee D., Chakrabarti A. (2014). Anti-kindling Effect of Bezafibrate, a Peroxisome Proliferator-activated Receptors Alpha Agonist, in Pentylenetetrazole Induced Kindling Seizure Model. J. Epilepsy Res..

[80] Porta N., Vallée L., Lecointe C., Bouchaert E., Staels B., Bordet R., Auvin S. (2009). Fenofibrate, a peroxisome proliferator-activated receptor-α agonist, exerts anticonvulsive properties. Epilepsia.

[81] Adabi Mohazab R., Javadi-Paydar M., Delfan B., Dehpour A.R. (2012). Possible involvement of PPAR-gamma receptor and nitric oxide pathway in the anticonvulsant effect of acute pioglitazone on pentylenetetrazole-induced seizures in mice. Epilepsy Res..

[82] Sun H., Huang Y., Yu X., Li Y., Yang J., Li R., Deng Y., Zhao G. (2008). Peroxisome proliferator-activated receptor gamma agonist, rosiglitazone, suppresses CD40 expression and attenuates inflammatory responses after lithium pilocarpine-induced status epilepticus in rats. Int. J. Dev. Neurosci..

[83] Yu X., Shao X.-G., Sun H., Li Y.-N., Yang J., Deng Y.-C., Huang Y.-G. (2008). Activation of cerebral peroxisome proliferator-activated receptors gamma exerts neuroprotection by inhibiting oxidative stress following pilocarpine-induced status epilepticus. Brain Res..

[84] Peng J., Wang K., Xiang W., Li Y., Hao Y., Guan Y. (2019). Rosiglitazone polarizes microglia and protects against pilocarpine-induced status epilepticus. CNS Neurosci. Ther..

[85] Wong S.-B., Cheng S.-J., Hung W.-C., Lee W.-T., Min M.-Y. (2015). Rosiglitazone Suppresses In Vitro Seizures in Hippocampal Slice by Inhibiting Presynaptic Glutamate Release in a Model of Temporal Lobe Epilepsy. PLoS ONE.

[86] Simeone T.A., Matthews S.A., Samson K.K., Simeone K.A. (2017). Regulation of brain PPARgamma2 contributes to ketogenic diet anti-seizure efficacy. Exp. Neurol..

[87] Toscano E.C.d.B., Vieira É.L.M., Portela A.C.D.C., Caliari M.V., Brant J.A.S., Giannetti A.V., Suemoto C.K., Leite R.E.P., Nitrini R., Rachid M.A. (2020). Microgliosis is associated with visual memory decline in patients with temporal lobe epilepsy and hippocampal sclerosis: A clinicopathologic study. Epilepsy Behav..

[88] Sharma A.K., Jordan W.H., Reams R.Y., Hall D.G., Snyder P.W. (2008). Temporal Profile of Clinical Signs and Histopathologic Changes in an F-344 Rat Model of Kainic Acid–induced Mesial Temporal Lobe Epilepsy. Toxicol. Pathol..

[89] Kim J.-E., Choi H.-C., Song H.-K., Jo S.-M., Kim D.-S., Choi S.-Y., Kim Y.-I., Kang T.-C. (2010). Levetiracetam inhibits interleukin-1β inflammatory responses in the hippocampus and piriform cortex of epileptic rats. Neurosci. Lett..

[90] Puttachary S., Sharma S., Verma S., Yang Y., Putra M., Thippeswamy A., Luo D., Thippeswamy T. (2016). 1400W, a highly selective inducible nitric oxide synthase inhibitor is a potential disease modifier in the rat kainate model of temporal lobe epilepsy. Neurobiol. Dis..

[91] Orihuela R., McPherson C.A., Harry G.J. (2016). Microglial M1/M2 polarization and metabolic states. Br. J. Pharmacol..

[92] Guo S., Wang H., Yin Y. (2022). Microglia Polarization from M1 to M2 in Neurodegenerative Diseases. Front. Aging Neurosci..

[93] Fan Y.-Y., Huo J. (2021). A1/A2 astrocytes in central nervous system injuries and diseases: Angels or devils?. Neurochem. Int..

[94] Liu J.-T., Wu S.-X., Zhang H., Kuang F. (2018). Inhibition of MyD88 Signaling Skews Microglia/Macrophage Polarization and Attenuates Neuronal Apoptosis in the Hippocampus After Status Epilepticus in Mice. Neurotherapeutics.

[95] Messaoudi M., Lalonde R., Violle N., Javelot H., Desor D., Nejdi A., Bisson J.-F., Rougeot C., Pichelin M., Cazaubiel M. (2011). Assessment of psychotropic-like properties of a probiotic formulation (*Lactobacillus helveticus* R0052 and *Bifidobacterium longum* R0175) in rats and human subjects. Br. J. Nutr..

[96] Savignac H.M., Kiely B., Dinan T.G., Cryan J.F. (2014). *Bifidobacteria* exert strain-specific effects on stress-related behavior and physiology in BALB/c mice. Neurogastroenterol. Motil..

[97] Hu P., Lu Y., Pan B.-X., Zhang W.-H. (2022). New Insights into the Pivotal Role of the Amygdala in Inflammation-Related Depression and Anxiety Disorder. Int. J. Mol. Sci..

[98] Tian P., O’Riordan K.J., Lee Y., Wang G., Zhao J., Zhang H., Cryan J.F., Chen W. (2020). Towards a psychobiotic therapy for depression: Bifidobacterium breve CCFM1025 reverses chronic stress-induced depressive symptoms and gut microbial abnormalities in mice. Neurobiol. Stress.

[99] Bercik P., Park A.J., Sinclair D., Khoshdel A., Lu J., Huang X., Deng Y., Blennerhassett P.A., Fahnestock M., Moine D. (2011). The anxiolytic effect of Bifidobacterium longum NCC3001 involves vagal pathways for gut-brain communication. Neurogastroenterol. Motil..

[100] Graham B.M., Daher M. (2016). Estradiol and Progesterone have Opposing Roles in the Regulation of Fear Extinction in Female Rats. Neuropsychopharmacology.

[101] Galeeva A.Y., Tuohimaa P., Shalyapina V.G. (2003). The role of sex steroids in forming anxiety states in female mice. Neurosci. Behav. Physiol..

[102] Arakawa K., Arakawa H., Hueston C.M., Deak T. (2014). Effects of the Estrous Cycle and Ovarian Hormones on Central Expression of Interleukin-1 Evoked by Stress in Female Rats. Neuroendocrinology.

[103] Kovalenko A.A., Zakharova M.V., Schwarz A.P., Dyomina A.V., Zubareva O.E., Zaitsev A.V. (2022). Changes in Metabotropic Glutamate Receptor Gene Expression in Rat Brain in a Lithium–Pilocarpine Model of Temporal Lobe Epilepsy. Int. J. Mol. Sci..

[104] Zubareva O.E., Kovalenko A.A.A., Kalemenev S.V., Schwarz A.P., Karyakin V.B., Zaitsev A.V. (2018). Alterations in mRNA expression of glutamate receptor subunits and excitatory amino acid transporters following pilocarpine-induced seizures in rats. Neurosci. Lett..

[105] Racine R.J. (1972). Modification of seizure activity by electrical stimulation. II. Motor seizure. Electroencephalogr. Clin. Neurophysiol..

[106] Paxinos G., Watson C. (2007). The Rat Brain in Stereotaxic Coordinates.

[107] Livak K.J., Schmittgen T.D. (2001). Analysis of Relative Gene Expression Data Using Real-Time Quantitative PCR and the 2^−ΔΔCT^ Method. Methods.

[108] Schwarz A.P., Malygina D.A., Kovalenko A.A., Trofimov A.N., Zaitsev A.V. (2020). Multiplex qPCR assay for assessment of reference gene expression stability in rat tissues/samples. Mol. Cell. Probes.

[109] Bercik P., Verdu E.F., Foster J.A., Macri J., Potter M., Huang X., Malinowski P., Jackson W., Blennerhassett P., Neufeld K.A. (2010). Chronic Gastrointestinal Inflammation Induces Anxiety-like Behavior and Alters Central Nervous System Biochemistry in Mice. Gastroenterology.

[110] Walsh R.N., Cummins R.A. (1976). The open-field test: A critical review. Psychol. Bull..

[111] Pellow S., Chopin P., File S.E., Briley M. (1985). Validation of open: Closed arm entries in an elevated plus-maze as a measure of anxiety in the rat. J. Neurosci. Methods.

[112] File S.E., Hyde J.R. (1978). Can social interaction be used to measure anxiety?. Br. J. Pharmacol..

[113] Bogdanova O.V., Kanekar S., D’Anci K.E., Renshaw P.F. (2013). Factors influencing behavior in the forced swim test. Physiol. Behav..

[114] Cernecka H., Doka G., Srankova J., Pivackova L., Malikova E., Galkova K., Kyselovic J., Krenek P., Klimas J. (2016). Ramipril restores PPARβ/δ and PPARγ expressions and reduces cardiac NADPH oxidase but fails to restore cardiac function and accompanied myosin heavy chain ratio shift in severe anthracycline-induced cardiomyopathy in rat. Eur. J. Pharmacol..

[115] Chistyakov D.V., Aleshin S.E., Astakhova A.A., Sergeeva M.G., Reiser G. (2015). Regulation of peroxisome proliferator-activated receptors (PPAR) α and -γ of rat brain astrocytes in the course of activation by toll-like receptor agonists. J. Neurochem..

[116] Raghavendra V., Tanga F.Y., DeLeo J.A. (2004). Attenuation of Morphine Tolerance, Withdrawal-Induced Hyperalgesia, and Associated Spinal Inflammatory Immune Responses by Propentofylline in Rats. Neuropsychopharmacology.

[117] Rioja I., Bush K.A., Buckton J.B., Dickson M.C., Life P.F. (2004). Joint cytokine quantification in two rodent arthritis models: Kinetics of expression, correlation of mRNA and protein levels and response to prednisolone treatment. Clin. Exp. Immunol..

[118] Bonefeld B.E., Elfving B., Wegener G. (2008). Reference genes for normalization: A study of rat brain tissue. Synapse.

[119] Lin W., Burks C.A., Hansen D.R., Kinnamon S.C., Gilbertson T.A. (2004). Taste receptor cells express pH-sensitive leak K+ channels. J. Neurophysiol..

[120] Yamaguchi M., Yamauchi A., Nishimura M., Ueda N., Naito S. (2005). Soybean oil fat emulsion prevents cytochrome P450 mRNA down-regulation induced by fat-free overdose total parenteral nutrition in infant rats. Biol. Pharm. Bull..

[121] Swijsen A., Nelissen K., Janssen D., Rigo J.M., Hoogland G. (2012). Validation of reference genes for quantitative real-time PCR studies in the dentate gyrus after experimental febrile seizures. BMC Res. Notes.

[122] Pohjanvirta R., Niittynen M., Lindén J., Boutros P.C., Moffat I.D., Okey A.B. (2006). Evaluation of various housekeeping genes for their applicability for normalization of mRNA expression in dioxin-treated rats. Chem. Biol. Interact..

[123] Malkin S.L., Amakhin D.V., Veniaminova E.A., Kim K.K., Zubareva O.E., Magazanik L.G., Zaitsev A.V. (2016). Changes of AMPA receptor properties in the neocortex and hippocampus following pilocarpine-induced status epilepticus in rats. Neuroscience.

[124] Cook N.L., Vink R., Donkin J.J., van den Heuvel C. (2009). Validation of reference genes for normalization of real-time quantitative RT-PCR data in traumatic brain injury. J. Neurosci. Res..

[125] Langnaese K., John R., Schweizer H., Ebmeyer U., Keilhoff G. (2008). Selection of reference genes for quantitative real-time PCR in a rat asphyxial cardiac arrest model. BMC Mol. Biol..

